# Celiac Disease and Targeting the Molecular Mechanisms of Autoimmunity in COVID Pandemic

**DOI:** 10.3390/ijms23147719

**Published:** 2022-07-13

**Authors:** Laura Marinela Ailioaie, Constantin Ailioaie, Gerhard Litscher, Dragos Andrei Chiran

**Affiliations:** 1 Department of Medical Physics, Alexandru Ioan Cuza University, 11 Carol I Boulevard, 700506 Iasi, Romania; lauraailioaie@yahoo.com (L.M.A.); laserail_mail@yahoo.com (C.A.); 2 Research Unit of Biomedical Engineering in Anesthesia and Intensive Care Medicine, Research Unit for Complementary and Integrative Laser Medicine, Traditional Chinese Medicine (TCM) Research Center Graz, Department of Anesthesiology and Intensive Care Medicine, Medical University of Graz, Auenbruggerplatz 39, 8036 Graz, Austria; 3 Department of Morpho-Functional Sciences I, Faculty of Medicine, Grigore T. Popa University of Medicine and Pharmacy, 16 Universitatii St., 700115 Iasi, Romania; dragos_chr@yahoo.com

**Keywords:** children, intestinal permeability, gluten, larazotide acetate, MIS-C, SARS-CoV-2, tight junctions, zonulin

## Abstract

Celiac disease (CD) comprises over 1% of the world’s population and is a chronic multisystem immune-mediated condition manifested by digestive and/or extradigestive symptoms caused by food intake of gluten. This review looked at the risk of children diagnosed with CD developing SARS-CoV-2 infection and possible severe forms of COVID-19. A better understanding of the interaction and effects of SARS-CoV-2 infection in CD is very important, as is the role of environmental and genetic factors, but especially the molecular mechanisms involved in modulating intestinal permeability with impact on autoimmunity. CD inspired the testing of a zonulin antagonist for the fulminant form of multisystem inflammatory syndrome in children (MIS-C) and paved the way for the discovery of new molecules to regulate the small intestine barrier function and immune responses. Original published works on COVID-19 and CD, new data and points of view have been analyzed because this dangerous virus SARS-CoV-2 is still here and yet influencing our lives. Medical science continues to focus on all uncertainties triggered by SARS-CoV-2 infection and its consequences, including in CD. Although the COVID-19 pandemic seems to be gradually extinguishing, there is a wealth of information and knowledge gained over the last two years and important life lessons to analyze, as well as relevant conclusions to be drawn to deal with future pandemics. Zonulin is being studied extensively in immunoengineering as an adjuvant to improving the absorption of new drugs and oral vaccines.

## 1. Introduction

A new coronavirus was first detected in Wuhan, China, in late 2019 and afterwards was termed severe acute respiratory syndrome coronavirus 2 (SARS-CoV-2). After the disorder, it became known as Coronavirus Disease-2019 (COVID-19) and led to the current pandemic and the global health crisis still present with major implications worldwide. World Health Organization (WHO) declared COVID-19 a pandemic in March 2020. Globally, as of 12 May 2022, over 516 million confirmed cases of COVID-19 and over six million deaths were reported; meanwhile, over 11 billion doses of vaccine have been administered [1,2,3,4,5].

Although the COVID-19 pandemic seems to be gradually extinguishing, there is a wealth of information and knowledge gained over the last two years and important life lessons to be analyzed, as well as relevant conclusions to be drawn for the future in all areas, but especially in molecular medicine and drug discovery, virology, epidemiology, genetics, immunology, vaccinology and clinical disciplines such as gastroenterology.

A recently published paper by Fasano [6] reaffirms the extraordinary perception of Hippocrates (460-377 Before Common Era, BCE), the father of modern medicine, who thousands of years ago postulated that “all disease begins in the gut” [7], which has only recently been recognized by the newest introspections in molecular and cellular pathophysiological mechanisms of myriad persistent inflammatory disorders that cause serious medical problems and burdens worldwide. Up until a few decades ago, until the elucidation of the human genome [6,8], the explanatory concepts were based on only two factors—genetic susceptibility and stochastic events triggered by surrounding circumstances—which formed the basis for modeling almost all conditions and even neoplasms, the current epidemiology has invalidated this model. Complete human genome decryption gave us limited knowledge, and the twenty-three thousand genes and the postulate of “one gene, one protein, one disease” cannot explain the intrinsic puzzle of health and diseases, and by no means the real explosion of persistent illnesses caused by inflammatory processes. This complex mutual interaction is controlled by many adjacent surfaces or interfaces between our organism and the ambient, from which the longest [about 6.7 to 7.6 metres (22 to 25 feet) long] [9], and the largest (the absorptive surface area is actually about 250 square meters, i.e., almost 2700 square feet—the size of a tennis court!) [10], is the human small intestine. The intestinal mucosa is responsible for the final interplay with the surroundings, i.e., the minute organisms producing disease (bacteria, viruses etc.), nutritive substances, waste materials that may contaminate, and so on. This third important player is intestinal permeability, which finely modulates the molecular transit between the tubular cavity of the small intestine and the layer of areolar connective tissue under the mucous membrane, balancing forbearance or immune reaction to foreign antigens, i.e., the autoimmunity [6].

Tight junctions (TJs) between cells are important controllers of antigen transit [11], being molecularly coordinated by zonulin, the only known modulator of intestinal permeability [6]. The activation of the zonulin pathway could be initiated even by short-term contact with the abundance of bacteria, viruses, gluten (for celiac disease) and others. The zonulin pathway is important for multiple molecular and cellular physiological mechanisms for keeping up mucosal homeostasis. The disruption of this pathway and epithelial and endothelial barrier functions, as well as the transformation of the constituents or activity of the intestinal microbiome, leads to many (but not all) chronic inflammatory or autoimmune diseases, such as celiac disease (CD), type 1 diabetes mellitus (T1DM), obesity, etc. [6].

The main goal of this review was to examine the risk of children diagnosed with CD contracting the SARS-CoV-2 infection and developing severe forms of COVID-19. The second aim was to provide a better understanding of the interactions and effects of SARS-CoV-2 infection in children and adolescents diagnosed with CD. The third purpose was to highlight the molecular mechanisms underlying CD and to analyze zonulin as a regulator of intestinal permeability in relation to a formidable pathology called multisystem inflammatory syndrome in children (MIS-C), which is triggered within a few weeks of viral contagion from contact or infection with SARS-CoV-2. This review reveals how CD in the COVID pandemic inspired the testing of an adjuvant drug for the fulminant form of MIS-C and paved the way for the discovery of new molecules.

Original published works on COVID-19 and CD and new data and points of view have been analyzed because SARS-CoV-2 remains and continues to influence our lives. The efforts of the scientific world continue to address the medical uncertainties triggered by the SARS-CoV-2 infection and its consequences, including in CD.

Tasks to face future pandemics are to develop new ways for quick and precision diagnosis and quantified management of infectious diseases by understanding the molecular mechanisms and how genes, proteins and other molecules interact within our cells.

## 2. Celiac Disease in Children—General Aspects

Celiac disease is a chronic inflammatory disease that primarily affects the small intestine following the ingestion of gluten and the related prolamins found in wheat, rye, oats, and barley. It has a prevalence in the general population worldwide of approximately 1% [12]. Within the last three decades, its prevalence raised due to more accurate diagnostic tests, and the age of diagnosis also increased from under 2 years to 6–9 years [13]. The Middle East, North Africa and India, once with low CD rates, have a higher prevalence [14] nowadays. CD is often underdiagnosed due to the heterogeneity of the clinical manifestations [15]. Most pediatric subjects experience “classic symptoms”: chronic diarrhea, steatorrhea, bloating, abdominal pain, irritability, and other signs of malabsorption, but few patients lack symptoms and are accidentally diagnosed [16]. Women are 1.5 times more affected, and gastrointestinal (GI) infections, use of antibiotics or proton pump inhibitors, and age when gluten was introduced into the diet represent environmental risk factors [17,18,19,20,21].

Genetic factors are recognized in the pathogenesis of CD; the human leukocyte antigen (HLA) class II (HLA)-DQ2 (allele DQA1 * 0501 and haplotypes DQB1 * 0201) and HLA-DQ8 (DQA1 * 0301 and DQB1 * 0302) or other variants are highlighted, but not sufficient to confirm or predict the onset of the disease [13,22,23]. Over 99% of CD patients have the HLA-DQ2 or HLA-DQ8 molecule, compared to only 40% of the general population [24].

In addition to genetic predisposition, contact with gluten, prolamins, the gluten-induced innate proinflammatory immune response, the tissue transglutaminase autoantigen (tTG), and other causes such as loss of intestinal barrier function, inadequate adaptive immune response and abnormal intestinal microbiome, may be involved in triggering the autoimmune process of CD. Anti-tissue transglutaminase (anti-tTG), formerly known as anti-tissue transglutaminase 2 (anti-tTG2), are autoantibodies of class IgA and IgG produced by tTG-specific/or tTG2 (old terminology)-specific B cells. In the past, the detection of anti-tissue transglutaminase 2 (anti-tTG2) antibodies in serum as important markers of CD, as well as the presence of other autoimmune phenomena, have included CD in the category of autoimmune diseases. Anti-tTG2 autoantibodies or newer, with the latest terminology—anti-tTG antibodies—are produced in the gut, where they are deposited much earlier before entering the general circulation [25,26,27].

Lack of breastfeeding and gluten intake before the age of six months and GI tract infections may increase the risk of CD [28,29]. Rotavirus vaccination can reduce the prevalence of CD in children and adolescents [30,31,32] since viral intestinal infections can alter the host’s local immune response for a very long time [33,34,35]. The role of bacteria, such as *Clostridium difficile*, *Helicobacter pylori* and *Streptococcus pneumoniae*, is not fully clarified in the pathophysiology of CD [36,37,38,39].

In 1950, Willem Dicke discovered that gluten from wheat is the key determinant of CD symptoms [26,40]. Over the decades, various theories have suggested that gluten would cause direct toxic damage to the lining of the small intestine [41,42,43], and because of its high resistance to the degradation by intestinal enzymes, it may increase the permeability of the intestinal mucosa [44,45,46,47]. Following this process, immunogenic gluten peptides cross the intestinal barrier and reach the general circulation, prolonging the inflammatory processes [48,49,50,51]. The penetration of undigested fragments of gluten peptides into intestinal lamina propria leads to their deamidation by the tissue enzyme transglutaminase 2 (TG2). This process of deamidation by TG2 is the cornerstone of CD pathophysiology, and anti-TG2 antibodies are used as biomarkers for positive diagnosis [43,52].

Deamidated native and gliadin peptides are taken up and presented to HLA-DQ2 and DQ8 molecules by dendritic cells (DC) and, via T-helper cells, will initiate an adaptive immune response. At the same time, α-amylase/trypsin inhibitors (ATI) and wheat lectins trigger the body’s innate immune response by stimulating the Toll-like receptor (TLR) 4 on myeloid cells and antigen-presenting cells (dendritic cells, monocytes, macrophages) in the intestinal mucosa, with the release of interleukins 8 (IL-8), 15 (IL-15), tumor necrosis factor-alpha (TNF-α) and monocyte chemoattractant protein-1 (MCP-1) [45,52,53,54,55].

Stimulated T-helper 1 (Th1) lymphocytes release IL-15, IL-21 and interferon-gamma (IFN-γ), which activate and promote cytotoxic intraepithelial CD8+ lymphocytes (IEL), facilitating the damage of the mucosa and intestinal wall. Activated T-helper type 2 (Th2) lymphocytes participate in the differentiation and activation of B lymphocytes, which stimulate the production of IgM, IgG and IgA, anti-TG2, anti-gliadin and anti-endomysium antibodies [56,57,58]. Some studies suggest that some gliadin peptides bind to TLR2 receptors and will influence the increase in IL-1 production through myeloid differentiation primary response 88 (MYD88), a key protein involved in the release of zonulin after gluten ingestion [59,60].

Although CD8+ cytotoxic cells and CD4+ Th1 cells are gluten-specific, and they are central exponents by releasing proinflammatory cytokines (IL-1β and IL-18), inflammatory pathways induced by cell death may also be involved in keeping the disease active by delivering proinflammatory molecules, such as the alarmins high-mobility group box-1 (HMGB1), IL-33 and IL-1α [61,62,63]. Helper T-cells produce proinflammatory cytokines (IFN-γ and TNF-α), which will further increase intestinal permeability and, in association with killer T-cells, trigger gluten enteropathy, which allows for intestinal retrotranscytosis of secretory IgA (SIgA)-gliadin complexes to act [64,65]. In the vast majority of individuals, all manifestations of the immune conflict disappear with a gluten-free diet [66].

A positive diagnosis of CD can be achieved through a combination of clinical parameters, immunological parameters (positive serological levels for total IgA and IgA anti-intestinal transglutaminase 2 antibodies (TGA-IgA), IgA anti-endomysium antibodies (EMA-IgA) and IgG deamidated gliadin peptide (DGP) antibodies (DGP-IgG) and/or histological data, obtained by biopsy; in clinical practice, the serologic IgA tissue transglutaminase antibodies have a sensitivity close to 97%, while EMA-IgA antibodies are highly specific markers (approximately 100%) for CD diagnosis [67,68,69,70,71,72,73].

In adults, a duodenal biopsy is the current gold standard for CD-positive diagnosis [74], whereas, in children, a biopsy is only needed when they have positive anti-tissue transglutaminase IgA antibodies (anti-tTG IgA) but with titers less than 10 times the upper limit of normal. For a positive diagnosis, they should have ≥4 biopsies of the distal duodenum and ≥1 of the duodenal bulb during a gluten-containing diet. A villus/crypt ratio < 2 indicates mucosal damage. In case of uncertain or discordant results between serology (TGA-IgA level) and histopathological appearance, a second opinion of an experienced histopathologist and/or a new biopsy is required [13,75].

Histopathological aspects (atrophy of the intestinal villi and crypt hyperplasia) are classified according to Marsh-Oberhuber criteria [76,77].

Current guidelines for positive CD diagnosis require four out of the following five criteria: (1) typical symptoms (diarrhea and malabsorption); (2) antibody positivity; (3) HLA-DQ2 and/or HLA-DQ8 positive; (4) histological intestinal lesions (atrophied villi and minor lesions); and (5) a clinically positive response to GFD [78,79]. European Society for Paediatric Gastroenterology Hepatology and Nutrition (ESPGHAN) and other recent studies recommend a diagnostic approach without biopsy, especially in children with T1DM and positive serological tests, even in the absence of symptoms [80,81,82].

Children with CD should be monitored in the first six months after diagnosis and then annually [83]. GFD helps resolve digestive and extra-digestive manifestations but can induce deficiencies in minerals and vitamins, together with psychological problems [84,85]. Determining adherence to a GFD can be heterogeneous due to gluten contamination, poor labeling, and restrictive dietary barriers [86,87,88].

In the CD population, there are two types of refractory forms; type 1 should be treated with a strict diet and oral budesonide, steroids in general, or in combination with azathioprine. Type 2 CD also benefits from steroids, associated or not with cyclophosphamide, cladribine, anti-TNF antibodies, and, if possible, stem cell therapy and transplantation. Patients with type 2 refractory form are at risk of developing T-cell lymphoma [79,89,90,91,92].

Significant progress has been made over the past decade to better understand the pathophysiology of CD, which has guided research directions for possible new treatments targeting junctions between intestinal lumen enterocytes, interfering with the inflammatory cascade to limit mucosal destruction or the invention of digestive enzymes with an intraluminal action and peptide binding agents that turn gluten into a non-toxic food [93].

To date, there is no Food and Drug Administration (FDA)-approved drug for the treatment of CD, and the only recommendation for reducing or eliminating the symptoms of this disease is to avoid consuming gluten-based products. There are several ongoing clinical trials testing pharmacological products for CD therapy. Currently, there are two advanced clinical trials testing the drugs: AT-1001 (Larazotide acetate) and IMGX-003 (Latiglutenase; formerly known as ALV003). These products are intended to relieve CD symptoms in two different ways. AT-1001 attempts to close or repair the defect of villi tight junctions, while IMGX-003 works as a gluten endopeptidase that breaks down gluten in the stomach before being absorbed in the small intestine [74].

Larazotide acetate is a synthetic peptide with eight amino acids, which works as a regulator of intestinal permeability by the antagonizing action of zonulin, a fundamental protein of the intestinal intercellular junction participating in the pathogenesis of CD. The other enzymatic treatment is IMGX-003 administered orally, which works from the stomach by fragmenting gluten and then in the small intestine, with the effect of improving the quality of life (QOL) and multiple symptoms induced by involuntary consumption of gluten [94].

Summarizing the data from the literature on CD management in children and adults, the following observations can be made: —at the current level of knowledge, the treatment and prevention of CD recurrence can be achieved only by suppressing gluten from the patient’s diet for life; —prevention of immune stimulation after absorption of gluten in the small intestine by immunosuppressive drugs (steroids, azathioprine, anti-cytokines, HLA-DQ2 blockers, cathepsin inhibitors, vaccines, etc.); —retention of gluten in the intestinal lumen by various polymers, antibodies, etc.; —prevention of absorption of digested gluten by a zonulin antagonist (e.g., Larazotide acetate); —use of tissue transglutaminase inhibitors; —reducing the immunogenic power of gluten through genetic, thermal, enzymatic engineering techniques, and so on.

## 3. Celiac Disease in Children during COVID Pandemic

Although COVID-19 primarily affects the respiratory system, many children also have GI symptoms manifested by cramps, abdominal pain, nausea, vomiting and diarrhea. Calitri et al. reviewed the pathophysiological mechanisms, clinical symptoms, diagnosis and management of COVID-19, and the impact of the disease on the digestive tract in children. GI manifestations appear to be more common in COVID-19 in children than in adult patients. The symptoms are usually of short duration and can be resolved by symptomatic treatment. However, in a small number of children, GI involvement precedes severe forms of the multisystem inflammatory syndrome in children (MIS-C). The SARS-CoV-2 virus should be tested in stool samples in sick children by real-time polymerase chain reaction (real-time PCR or RT-PCR) (a laboratory technique of molecular biology based on the polymerase chain reaction). In children at risk, such as those with CD, intestinal inflammation, and chronic liver disease, COVID-19 does not appear to be more severe than in other patients on immunosuppressive therapy. Monitoring CD patients must be adapted to the pandemic to avoid unnecessary endoscopic examinations and duodenal biopsies. Telemedicine can be a good alternative for educating and monitoring chronic patients, keeping away from the risk of unwarranted interventions and viral transmission [95].

Because a CD outburst has been seen as a hallmark after SARS-CoV-2 infection, Cakir et al. pursued to study of the impact of the COVID-19 pandemic on the incidence and the clinical symptoms of CD. Researchers divided CD patients into two groups [diagnosed in pre-pandemic (January 2008–February 2020) and in pandemic period (March 2020–June 2021)] and compared them so as to reveal the differences between the groups regarding the clinical and the histological data. Supplementary information was gathered concerning the second subgroup (*n* = 22) diagnosed with CD and COVID-19 during the pandemic. It came out that the number of patients per year (12.1–37.6) and the percentage of patients who were diagnosed with CD increased during the pandemic (2.2% vs. 10%). T1DM has been reported in 17% of patients with CD, compared with only 4% before the pandemic. The incidence of moderate-severe mucosal lesions has been reduced by almost half in the pandemic (42.4% vs. 81.7%). More than one-third (36.3%) of patients diagnosed with CD during the pandemic had a previous severe infection with SARS-CoV-2, reflected by the biological markers and clinical symptoms. Authors accept as true that the incidence of CD and its association with T1DM increased in children during the COVID-19 pandemic [96].

In a recently published article on COVID-19 and CD, Trovato et al. reveal a new pathogenetic hypothesis regarding the outbreak of CD in the current pandemic, highlighting the role of COVID-19 as a potential trigger for celiac disease in predisposed patients. The context cited by the authors is the growing body of available information corroborated with the gut tropism with ciliated cells and intestinal enterocytes as ideal targets for the SARS-CoV-2 virus through high levels of angiotensin-converting enzyme 2 (ACE2) receptors and transmembrane serine protease 2 (TMPRSS2) expression. The virus can easily enter the cells by binding to ACE2, followed by its priming by TMPRSS2 and the activation and increase of inflammation locally. As an extra factor, priming of the spike protein by the serine protease TMPRSS2 in ciliated cells and the brush border of gut enterocytes is essential for SARS-CoV-2 to invade the cells of the intestinal lining, provoking mucosal deterioration, and conducting to increased permeability due to damage of the gut barrier. Further consequences are the movement of microbes, including microbial-associated molecular patterns (MAMPs), generating an inflammatory immune response by especially the macrophages and adipocytes as TLR-expressing cells of the mesentery fat, which on this path can extend into the systemic circulation. This complex portrayal reinforces the assumption that gut cells could contribute to an increase in the presence of the SARS-CoV-2 virus in the blood (higher viremia). A consequence of damage to the intestinal epithelium is a greater barrier permeability, which grants permission to the gliadin to pass into the intestinal lamina. However, loss of intestinal barrier function is very important in the pathogenesis of CD because it is a systemic autoimmune disease acquired by genetically predisposed subjects due to gliadin, which passes from the intestinal lumen to the lamina propria—either by crossing the barrier or by transcellular transfer. This transition is the first step toward disease progression because the binding of DGP to antigen-presenting cells (APC) takes place in the lamina propria. Based on these pairs of pathogenetic implications that may precede the onset of CD, the authors concluded that genetically predisposed patients are more likely to develop CD after SARS CoV-2 infection, so the current pandemic could be a potential trigger for an outburst of CD in the very near future [97].

Asri et al. examined the levels of genes that influence immune homeostasis and are related to inflammation [IL-6, CD4, CD25 and forkhead box P3 (FOXP3)] in peripheral fresh whole blood samples from 60 newly diagnosed CD people (mean age 35.40 ± 24.12 yrs.), 30 patients with severe COVID-19 (mean age 59.67 ± 17.22 yrs.), and 60 healthy subjects (mean age 35.6 ± 13.02 yrs.), enrolled for a period of 6 months in 2020. RNA expression levels of the aforementioned genes were evaluated applying real-time quantitative RT-PCR and performant statistical analysis. Higher expression of CD4, CD25 and FOXP3 was determined in patients with CD compared to the control group and the COVID-19 group, the latter having lower expression levels compared to controls. However, a higher expression of IL-6 was observed in both groups of patients compared to controls. Authors concluded that due to the high expression of IL-6, patients with untreated CD may be at higher risk of developing severe COVID-19, but the increased expression of anti-inflammatory markers may be salutary for them, possible through diminishing the gravity of COVID-19, aspects to be scientifically proved in time to come research on CD patients contaminated with SARS-CoV-2 [98].

Renzo et al. managed an online analysis of many Italian pediatric centers engaged in GI endoscopy in order to assess the adjustments of this medical branch in the COVID-19 pandemic during high viral transmission. Facts and statistics collected for analysis were compared in two selected periods. Findings of the study from 24 pediatric endoscopy units that responded highlighted a marked decrease in GI endoscopy procedures with a total reduction of 37.2% in 2020 compared to 2019, consistent with another new survey conducted in 12 European centers in April 2020. There has been a dramatic drop from 621 to 279 (55.1%) from 2019 to 2020 in the procedures for prepositive CD, with the longest waiting lists for the new onset of CD. All centers had to suspend or reschedule the GI endoscopies due to the outbreak of SARS-CoV-2 infections, especially since mid-March’20. Therefore, it could be concluded that the effect of COVID-19 on the practice of GI endoscopy in Italian children was important [99].

Although SARS-CoV-2 virus infection is transmitted mainly by tiny drops, in addition to respiratory manifestations, patients often have GI symptoms and liver damage. Concas et al. published an updated practical review for gastroenterologists facing many patients with COVID-19 and chronic GI disease (inflammatory bowel disease, celiac disease, chronic liver disease). Rapid collecting of valuable information from the latest publications for improved standards in medical consultation and patient care regarding COVID-19 is essential. The authors explored all available medical references, deepened the origin and pathophysiological mechanisms of COVID-19, examined the clinical manifestations of GI involvement, introduced the latest guidelines on main practical GI procedures and recommended immunosuppressive therapy, and emphasized the importance of maintaining social distance. Particular attention should be paid to fecal-oral transmission and intestinal microbiota in COVID-19. In general, patients with CD are not usually considered immunocompromised, except for those with an extremely poor diet and weight loss, refractory CD type 2, immunosuppressive drugs, or other serious illnesses that could develop severe COVID-19, and they must be under medical supervision. However, the authors pointed out that as of the time of their analysis, no previous research had shown that patients with CD would be at increased risk of developing severe COVID-19, but important data are collected continuously in an international registry (SECURE-Celiac) in which clinicians worldwide are asked to report all cases of COVID-19 in celiac patients, regardless of the severity of the disease. A review of studies conducted by authors on publications from China, Italy, the United States, and the United Kingdom, having samples from children and adolescents in the relationship between celiac disease and SARS-CoV-2 infection, showed that a small percentage did COVID-19, with possible arguments the active resistance of children to the virus or multiple unrecognized asymptomatic cases. Although with a mild course of COVID-19 compared to adults, preschool children (under five years) had a higher load of viral RNA in the nasopharynx than other ages and adults. Regarding the presence of GI symptoms in children with COVID-19, in any case of the severity of the disease, it is essential to note that stool samples and rectal swabs may be positive for viral RNA for several days after infection, and children appear to eliminate the virus a longer time than adults, which makes them possible essential viral transmitters [100].

In a research correspondence on the results of COVID-19 in CD, Uche-Anya et al. conducted an investigation on the International Registry of Celiac Disease Patients, SECURE-CELIAC registry, and they found 12% hospitalization and 2.5% mortality rates, reflecting the low risk of admission to hospital for treatment, or death. In conclusion, according to this research, patients with CD do not have an increased risk of hospitalization or death due to COVID-19, but old age and new GI symptoms during SARS-CoV-2 infection may trigger an unfavorable course [101].

Mehtab et al. assessed the consequences of lockdown and limited mobility during the COVID-19 pandemic on the accurate adhesion to GFD, symptom management and QOL in CD patients from northern India. Researchers sent a web-based questionnaire to 3130 patients on WhatsApp and contacted telephonically 68 patients, who were not present on any social network, and finally included in the analysis 505 fully responders. The questionnaire comprised both specific purpose and certified questions, introduced after the review of the medical literature, discussions and seminars with experts, and included the CD adherence test, the celiac symptom index score and CD-relevant QOL. Of the 505 patients finally included, 6.7% had had poor GFD compliance before the pandemic, but their number nearly doubled during the pandemic. In addition, almost 5% were diagnosed with CD when tested for SARS-CoV-2 infection. About two-thirds of patients liked the online consultation more than in person. Most usual problems to overcome during the lockdown were high delivery prices for gluten-free (GF) food at home (54.4%), an increase in prices for regular GF food (43.1%) and long-distance travel to get GF food (44.9%). In conclusion, as a positive effect, the pandemic paved the way for teleconsultation for patients with CD but negatively affected the GFD, symptom management, and the QOL due to lack of money at home, high costs for the purchase and delivery of GF food at home, sometimes very difficult to find. Future steps should be taken to maintain GF food supply chains, online consultation and monitoring of CD patients in case of regional lockdowns or across the country [102].

Falcomer et al. investigated the effects of the pandemic as an extra burden on long-term celiac disease (CD), which further compromised the QOL of patients diagnosed with this condition in Brazil. The purpose of this research was to assess the QOL of Brazilian patients with CD during the current pandemic caused by the SAR-Cov-2 epidemic and its very fast dissemination around the globe, subsequent restrictions and lockdowns, and the overlapping dietary restrictions and other overloads on CD patients. The study was conducted online across the country through a previously validated questionnaire in Brazilian and Portuguese to investigate the QOL of patients diagnosed with CD. The answers to the sent and self-administered questionnaire were received from 674 patients with CD and revealed the following aspects: QOL of people with CD in Brazil has not been negatively affected by the current pandemic; GI manifestations had the greatest influence, followed by social ones; unlike psychological, mental suffering or affective, with no effect on the QOL of CD people in Brazil; all other issues related to profession, age, gender, marital status, children, or even a positive test for COVID-19 did not affect QOL in CD subjects; most dramatic influence on QOL had non-compliance to GFD and the use of drugs to prevent or relieve mental depression in CD patients. The authors believe that further research is needed to extend these results to the post-pandemic COVID-19 era [103].

In another article published by Monzani et al., the authors studied GFD adherence during COVID-19 lockdown in Italian patients with CD (adults and children/adolescents) using an online survey. Out of the total of 1983 replies, 369 (18.6%) were for children/adolescents with CD (answers given by their parents or caregivers), and the remaining 81.4% for adults with CD. GFD adherence was unchanged in 70% of children (69% of adults, respectively) and improved for 29% in both age groups. The authors reported that the particularities that increased the likelihood of reporting better adherence in adults were the constant appearance of CD symptoms in the last year before lockdown, but also usual partial compliance and testing of new natural, gluten-free formulas with more ingredients than usual. In the case of children or adolescents with CD, the critical factors were the existence of CD symptoms in the last year, CD antibodies yet positive, and also the existence of other family members diagnosed with CD. The conclusion was that the lockdown resulted in improved compliance to GFD in 33% of participants, but especially in those with poorer disease control, slaughtering sources of contamination or deviation, and new confidence in naturally gluten-free products [104].

In a recently published study, Temsah et al. analyzed the success of facts, information and skills acquired by parents and caregivers, as well as their views on unintended and preventable injuries, the well-being and safety of their children or adolescents during the COVID-19 pandemic lockdowns. Pre-post investigative research with predetermined assessments, such as questions about the socio-demographic status and knowledge acquisition before and after participating in a security campaign on 308 volunteer parents in Saudi Arabia, showed an improved score from 36.2 to 79.3, as well as an increased perception of the general expertise and accomplishments towards the security of children and adolescents, so that during lockdowns, additional training tools, programs and other pertinent information are justified to promote safe and harmless practices to parents, nurses, social workers and others [105].

Barschkett et al. compared the number of outpatient visits of children in the German national registry before and during the first wave of the COVID-19 pandemic, i.e., between January 2019 and June 2020. There was an 18% decrease in the number of outpatient visits per child during the first wave of the pandemic, with a significant decrease (51%) in intercurrent infections, especially in young children under five years, but for chronic diseases, the outpatient visits diminished only to a minor degree, for example, T1DM (to 92%), CD (to 86%) and hay fever (to 95%), as well as for mental and behavioral conditions have shown only insignificant differences. The authors concluded that lack of contact between children seems to reduce the transmission of infections. Future targeted educational and counseling measures, as well as adequate preventive measures to reduce stress and improve the QOL in children, are necessary and welcome, including for parents who have lost precious time away from work [106].

In a recently published paper, Dipasquale et al. investigated the effects and consequences of the COVID-19 pandemic in highlighting the nature and all the complications of the GI diseases in children and adolescents for a correct diagnosis and management in the COVID era. In this review, the authors turned their attention to pediatric gastroenterologists to assist in the correct diagnosis and management so that researchers presented evidence of digestive and clinical involvement of COVID-19; they highlighted the effects of COVID-19 on the clinical approach in children and adolescents with pre-existing disease or in an early stage from onset; and they also focused on the duty and restricted access to instrumental investigations, for example, endoscopy of the digestive tract in the coronavirus pandemic. It is currently unknown whether immunosuppressive therapy for inflammatory bowel disease (IBD) or chronic hepatitis is at risk and may cause adverse reactions in some subjects. In the case of patients in remission, outpatient follow-up consultations may be postponed, but telemedicine is especially recommended in the absence of any risk of infection. Any new therapies must be individualized, and there will be presented not only the benefits but especially the risks to each patient and the family. Psychological counseling therapy should be initiated for all children with chronic diseases and for their parents or caregivers. All endoscopic procedures that are not urgently needed or optional may be suspended while minimizing the risk of viral spread. Social distance and the use of individual safeguarding equipment should be further recommended, as well as SARS-CoV-2 vaccination [107].

Bükülmez et al. investigated the influence of COVID-19 on pediatric patients diagnosed with CD in Turkey. The authors tried to sound the alarm for the parents and caregivers of children diagnosed with CD to make them aware of the need for necessary measures to be taken in the current coronavirus pandemic for their children. COVID-19 has caused unpredictable changes in life through restrictions, lockdowns, severe complications and death in some cases. Several patients with CD have asplenism or hyposplenism and a higher risk of pneumococcal sepsis in the latter case. The authors designed and conducted a cross-sectional study between May and July 2020 through an online survey of a sample of 73 parents whose children with a mean age of 11.36 ± 4.36 years had confirmed CD diagnostic at a university hospital in Turkey. The most important results were that 90.4% of participants responded that SARS-CoV-2 infection was transmitted through small droplets from the respiratory tract released by sneezing, coughing, speaking, or after contact with virus-infected surfaces followed by touching the face. The vast majority (78.1%) said they had no problem following the GFD because they found all the GF foods needed. Parents of children with CD did not know that the risk of infection with this virus in their children may differ from that of healthy children, so the study accentuated that these parents should have been better informed about COVID-19. Parents also noticed the increase in the level of anxiety in their children during this pandemic, as well as the fact that they gained several extra pounds in lockdown, with a negative effect on their health, well-being and keeping up a healthy way of living [108].

In a letter to the editor of *Clinical Gastroenterology and Hepatology*, Lionetti et al. expressed interest in investigating whether the risk of coronavirus disease 2019 (COVID-19) is increased in children and adolescents with CD including the morbidity and mortality in these patients. The authors pointed out that Italy had a sudden spread of SARS-CoV-2 infection and that the crisis had hit the country hard, especially in the central part where there was an important center for children with CD, so the researchers took the opportunity to study the prevalence and the severity of COVID-19 in children with CD and compared them with general population data. Authors who signed the letter investigated between February and June 2020, through a telephone survey, using a questionnaire with 26 questions, the prevalence and clinical characteristics of SARS-CoV-2 infection in CD patients. The researchers included all children diagnosed with CD according to ESPGHAN criteria in a CD group who tested positive for SARS-CoV-2 infection. The control group was like this group but consisted of patients with possible COVID-19-related symptoms but untested. For the prevalence of COVID-19 in children and adolescents in the Marche region during the same period, the reports of the Italian National Institutes of Health and of the regional government were used. Of the initial 419 patients with CD contacted, only 387 had a positive response, of which 37% were males, with a mean age of 9.9 (1–16 years) and a mean age at CD diagnosis of 7.5 years (range of 6 months to 16 years). Of the total number of patients with CD, none had COVID-19 confirmed by laboratory testing, so the prevalence of COVID-19 was 0/387 (95% confidence interval, 0.0000–0.0095). 3.9% of patients (*n* = 15) had a fever but had no other symptoms associated with COVID-19. 5.9% of patients (*n* = 23) were included in the COVID-19 like- group (nine with fever and cough; two with fever, vomiting and diarrhea; ten with diarrhea and/or vomiting; two with cough), but none did not have respiratory failure or pneumonia and did not need oxygen administration or hospitalization. In that region, the confirmed prevalence of COVID-19 at 0–16 years was 155/199,289 (0.08%; 95% confidence interval, 0.0007–0.0009). Thus, the authors concluded that children with CD did not significantly increase the prevalence of COVID-19 compared to the general population, and children in the COVID-19 group did not develop the severe or complicated disease. As an observation, the authors noted that the number of infections could be underestimated in the CD group because they could not assess the number of all asymptomatic carriers of SAR-CoV-2, but the same limitation should be considered in the general reference population. The findings of this study are consistent with the conclusions of other studies that have shown that pediatric patients with CD do not have an increased risk of SARS-CoV-2 infection. However, COVID-19 has caused an unprecedented crisis in the global health system, forced the rethinking of the management of patients with acute or chronic diseases and opened wide the doors to telemedicine, which is very suitable for tracking CD patients. Although these findings are not of particular concern to CD, compliance with prevention measures in the general population is also necessary for this chronic disease, and other long-term studies may imply a better understanding of the risk of contracting COVID-19 in pediatric patients with CD [109].

In correspondence to the publisher of *Digestive and Liver Diseases*, Catassi et al. wrote about life-threatening delays in diagnosing CD due to COVID-19 lockdown in Italy as a very dark side of pandemics. Forced lockdown of the COVID-19 pandemic has had major consequences for primary care, even for habitual GI disorders, but sometimes severe or critical. For example, the authors succinctly outlined the history of one critical clinical case admitted to their regional medical academic center. A 17-month-old girl (breastfed only for four months and afterwards weaned with formula, cereals, meat and vegetables) was admitted during the Italian lockdown in March 2020 for abdominal pain, distention, and widespread edema. The weight and height were 8.0 Kg (below 3rd centile) and 70 cm (below 3rd centile), respectively. Laparotomy was applied for reduction of involved intestinal segments, but the day after surgery, the child was irritated and with significant edema of the face, abdomen, and upper and lower limbs. Laboratory data were normal, except for low serum albumin (2.8 g/dL) and total calcium (8 mg/dL). Because the clinical history suggested CD, the serum CD autoantibodies were measured, as suggested by the ESPGHAN diagnostic guidelines, and a GFD was immediately started before the results were obtained due to the gravity of the clinical manifestations. Diagnosis of CD was strongly suggested by high-level positivity (>10× UNL) of anti-DGP IgG and uncertain levels (1× UNL) of anti-tTG IgA. After 10 days of GFD, the edema had disappeared. CD diagnostic was clearly certified by the intestinal biopsy highlighting drastic villous atrophy and many intramembranous lymphocytes. After a month of GFD, the little girl continued to show signs of obvious improvement in her health. The authors concluded that this example is just one of many potentially life-threatening delays in the diagnosis and treatment of CD in children during the COVID-19 pandemic [110].

## 4. Molecular Mechanisms of SARS-CoV-2 Infection and How CD Led to an Adjuvant Drug for MIS-C

Management of COVID-19 is a constant challenge in the presence of Omicron’s mutations, which made it the most infectious coronavirus variant yet, and because effective treatments are not yet available worldwide, especially in severe forms, for example, in MIS-C. Coherent strategies are still needed to support, predict results and deal with new cases around the world. Therefore, it is vital to understand the complex molecular mechanisms of COVID-19 pathogenesis, especially in children and adolescents who developed fulminant cases of MIS-C, as well as in other autoimmune diseases, such as CD. It was noticed that a few weeks after contacting the SARS-CoV-2 virus responsible for triggering COVID-19 disease, even asymptomatic, some children or adolescents develop MIS-C if they have the virus that causes COVID-19 or they have been in contact with someone diagnosed with COVID-19. This disease, initially also called pediatric inflammatory, multisystem syndrome (PIMS), temporally associated with SARS-CoV-2 infection (PIMS-TS) or systemic inflammatory syndrome in COVID19 (SISCoV), is a systemic disease with incessant fever and maximum inflammation, which can be life-threatening because it can trigger multiple organ failure, even cardiogenic shock with ventricular dysfunction, and the family must seek medical attention immediately as most children will need intensive care. MIS-C is a severe consequence of COVID-19 in children or adolescents, connected with important hemodynamic, cardiovascular and other organs’ inflammation and damage, such as the lungs, kidneys, brain, skin, eyes, and marked GI symptoms. 

The critical onset of MIS-C is in a patient under the age of 21, with a high fever for at least 24 h, with inflammation proved by elevated inflammatory markers, hypotension, multisystem organ implication, and proof of SARS-CoV-2 infection on RT-PCR, antibody testing, or contact to persons with COVID-19 in the past 4–6 weeks, which is an immune activation syndrome with a cytokine storm that requires intensive care management. MIS-C can be a severe, even fatal, condition. Regardless of the severe presentation, most disturbances will resolve within a few weeks with intensive care, but severe cardiac implications will need a considerable rehabilitation period. Researchers are making a continuous effort to broaden their knowledge of the pathophysiology of MIS-C day by day, but medical doctors have continued to apply prudent management to these patients until all the molecular and clinical aspects of MIS-C and its long-term consequences are fully comprehended so they can develop globally accepted general clinical solutions for the treatment of these critically ill patients with MIS-C [111,112,113,114,115,116,117].

In 2020, Consiglio et al. applied systems-level analyses of blood immune cells, cytokines and autoantibodies, comparing four groups, as follows: children diagnosed with MIS-C, children infected with SARS-CoV-2, children with Kawasaki disease (KD) prior to COVID-19 era, and healthy children. The authors noted that the inflammatory response in MIS-C differs from the “cytokine storm” in severe COVID-19 cases; it has many elements in common with KD. However, it differs from KD in terms of the activated T-cells subset and IL-17A (which pushes forward the cytokine storm in KD, but not in MIS-C), the different biomarkers from arterial lesions, but especially through multiple autoantibodies with pathogenic potential in the pathophysiology of MIS-C, molecularly distinct and through the immune profiles of all other diseases analyzed. The authors reported high levels of autoantibodies that attack endoglines (glycoproteins expressed by endothelial cells responsible for arterial integrity) in a couple of cases with MIS-C and in a small number of patients with KD [118].

In a recent multicenter, retrospective cohort study published in Lancet Rheumatology, the authors analyzed the serum and plasma samples collected from 21 patients with MIS-C who were seropositive or PCR-positive (or both seropositive and PCR-positive) for SARS-CoV-2, with one exception (reported contact), treated at five clinical centers in Germany and Spain, with multiple control groups, as follows: asymptomatic or mild COVID-19 (*n* = 146 patients), KD (*n* = 24), systemic juvenile idiopathic arthritis (sJIA) in remission (*n* = 10), non-inflammatory patients with suspected growth retardation (*n* = 33), and 462 healthy controls. All samples from MIS-C patients, with two exceptions, and all samples from KD patients were collected and analyzed before intravenous immune globulins (IVIGs) administration. Results found autoantibodies against IL-1Ra together with a hyperphosphorylated isoform of IL-1Ra in most patients with MIS-C. Despite the reduced number of enrolled MIS-C subjects, many control patients both with inflammatory and non-inflammatory pathologies have proved the appearance of autoantibodies in MIS-C, obviously connected and relevant to, and potentially triggering, the hyperinflammatory condition characteristic of this disease [119].

Dhaliwal et al. analyzed the possible immunopathogenic mechanisms assumed comparatively in Severe Acute Respiratory Syndrome (SARS), Middle East Respiratory Syndrome (MERS), COVID-19, MIS-C and KD. They pointed out that SARS-CoV-2 is characterized by a modified spike polypeptide (S1) with a higher binding affinity to host NRP1 receptors, being responsible for high infectivity and tissue tropism in SARS-CoV-2 infections. The molecular mechanisms of COVID-19 appear to have delayed IFN responses, which are responsible for the high secretion of IL-6, IL-7 and TNF-α, hyperinflammation and worsening of clinical status, with the release of the following autoantibodies to dual specificity mitogen-activated protein kinase kinase 2 (MAP2K2), and to the casein kinase family (Casein kinase 1, alpha 1 (CSNK1A1), Casein kinase 2, alpha 1 (CSNK2A1) and Casein kinase 1, epsilon 1 (CSNK1E1)), found exclusively in MIS-C [120].

Studying the immune mechanisms in the pathophysiology of MIS-C, Gruber et al. highlighted the role of autoantibodies in attacking the endothelial, gastrointestinal, and immune cells. The authors stipulated that the anti-SARS-CoV-2 antibody collection in MIS-C is similar to a response in the recovery phase, i.e., the returning to health after infection. The type of cytokines denotes inflammation (IL-18 and IL-6), lymphocytic activation and myeloid chemotaxis [Chemokine (C-C motif) ligand 3 (CCL3), Chemokine (C-C motif) ligands 4 (CCL4), and CUB domain-containing protein 1 (CDCP1)], as well as a mucosal immune disorder [IL-17A, Chemokine (C-C motif) ligand 20 (CCL20), and Chemokine (C-C motif) ligand 28 (CCL28)]. Mass cytometry analysis indicated the activation of immune cells and their peripheric extravasation to affected tissues. There was also a decrease in non-classical monocytes and in different subsets of NK and T lymphocytes. The profile of autoantibodies was very complex, including the disease-connected autoantibodies but also the new ones that recognize endothelial, gastrointestinal, and immune-cell antigens. The autoantibodies in MIS-C target the most important organs, such as the heart, kidney, brain, GI tract and so on [121].

In SARS-CoV-2 infection, complement (C) activation is very important and has been histopathologically proven by endothelial deposition of complement, as well as by finding elevated serum C5a levels in severe forms of COVID-19. In both MIS-C and severe COVID-19, high serum concentrations of soluble C5b-9 (sC5b-9) have been identified in association with microangiopathy [122].

Bartsch et al. studied the differences in the severity of COVID-19 disease between adults and children by analyzing the humoral immune response of 60 adults with acute COVID-19 (26 severe, 34 mild), 25 children with mild SARS-CoV-2 infection, and 17 children with confirmed (*n* = 14) or suspected PCR or serology (home contacts, *n* = 3), who developed MIS-C (11 severe, 6 mild). In this research, the authors proved that low IgA and phagocytic activity accompany mild disease both in children and adults, and the disease severity is reflected by an increased distinct humoral immunity to COVID-19 pathology. There are specific changing patterns of SARS-CoV-2 IgA antibodies and the persistence of dysregulated and pro-inflammatory antibody profiles, which could be an indication of severe MIS-C. In spite of the fact that IgA could merely signify a biomarker of increased viremia in the lungs, it is presumed to have an important role in the mucosal immune barrier. Preservation of elevated levels of monocyte-activating pathogen-specific IgG, hyperphagocytosis with cytokine storm, T-cell activation and the exacerbation of inflammation predict a severe MIS-C [123].

In 2021, a US multidisciplinary team led by pediatric pulmonologist Yonker teamed up with Alessio Fasano, the pediatric gastroenterologist and researcher who discovered zonulin, the protein responsible for regulating intestinal tight junctions (TJs), published a study on the mechanisms that could be responsible for MIS-C, a rare and sometimes fatal post-COVID-19 complication. Yonker et al. analyzed samples from 100 children, divided into three groups: 19 patients diagnosed with MIS-C, 26 patients with acute COVID-19, and 55 witnesses. The fecal samples were evaluated for SARS-CoV-2 by RT-PCR, and plasma was explored for markers of mucosal barrier integrity, among which were zonulin. The authors demonstrated the presence of SARS-CoV-2 in the GI in most patients with MIS-C a few weeks after infection or initial exposure to the virus by measuring SARS-CoV-2 RNA in stool samples, the results suggesting the presence of an outbreak of continuous infection, the source of complications in MIS-C. Physiologically, the integrity of the intestinal mucosal barrier should stop the passage of viral antigens, toxins, or other substances given off by the SARS-CoV-2 virus from the lumen into the bloodstream [124].

Zonulin, as a modulator of intestinal permeability and its expression in CD, can induce reversible intestinal TJs’ disassembly between the adjacent epithelial cells and a subsequent increase in intestinal permeability, exactly as in the acute phase of CD, in which TJs are opened, and the permeability is increased [125].

In the above-mentioned research conducted by Yonker et al., the immune responses were measured with an ultrasensitive SARS-CoV-2 antigenemia probe for plasma samples from patients. The authors showed that the increased permeability of the mucosal barrier triggered by zonulin matched well with the SARS-CoV-2 antigenemia (SARS-CoV-2 spike protein, especially S1 region, and nucleocapsid antigens identified in the plasma of patients with MIS-C, a few weeks after infection or initial contact with SARS-CoV-2), which was significantly increased in patients with MIS-C compared to healthy controls or children with acute COVID-19. So, zonulin, as a biomarker of intestinal permeability, which allowed the ulterior flux of SARS-CoV-2 antigens into the circulating blood, generated the hyperinflammatory condition. In patients with MIS-C, it has been identified the cytokine storm with high concentrations of IL-1β, IL-6, IL-10, TNF-α, and especially IFN-γ, as an antiviral cytokine, comparatively to healthy controls or the children with acute COVID-19. The levels of IgM, IgG and IgA against the spike protein, or S1 region, were very high in plasma MIS-C patients. As an early adaptive immune response, anti-spike IgM levels were highest in acute COVID-19 cases and remained much higher than predicted, with a slow downward slope in the weeks after initial infection or exposure to SARS-CoV-2. The highest anti-spike IgG, anti-S1 IgG, and anti-RBD (anti-receptor binding domain) IgG were in patients with late-onset MIS-C. As the exponents of mucosal immunity, the levels of anti-spike IgA, anti-S1 IgA and anti-RBD IgA were very high and remained so for months in patients with MIS-C, reflecting the persistence of the virus. The authors concluded that the MIS-C immunoprofiles reflected continuous mucosal exposure to SARS-CoV-2. As evidence of their hypothesis, the medical doctors administrated to a MIS-C patient a zonulin antagonist, i.e., larazotide, and checked up on the antigenemia and the clinical outcome. The patient with MIS-C managed with larazotide experienced a significant simultaneous reduction in both plasma SARS-CoV-2 antigen concentrations, and in the inflammatory biomarkers, with a good clinical outcome superior to all existing treatments. Although the approach to the pathogenesis of MIS-C has been mechanistic, the authors open new perspectives for diagnosis, therapy and prevention in severe COVID-19 pathology in children, i.e., in MIS-C [124].

In both CD and SARS-CoV-2 infection, the common denominator is the intestinal barrier that leads to what is known as “leaky gut syndrome”. From an anatomopathological point of view, the small intestine is histologically structured in a single simple epithelial layer that plays an important role in the absorption of water and nutrients brought by intestinal peristalsis but also functions as a protective barrier system against pathogens. Inside the small intestine, the cellular epithelium is arranged in the so-called epithelial protrusions known as finger-like intestinal villi, which form most of the epithelial surface, where the absorption activity takes place. The intestinal villi are structured by epithelial cells with an absorbent role, mucus-secreting calyx and hormone-secreting enterocytes. The spaces between the villi are called crypts and have Paneth cells in their structure. These cells, discovered by Josef Paneth in the late 19th century, are pyramidal cells distributed between crypt base columnar (CBC) stem cells at the base of Lieberkühn’s crypts in the small intestine epithelium and act as “assisting cells” in innate intestinal immunity. Paneth cells exhibit on their surface crucial constituents of important signal transduction pathways, such as Notch delta-like ligands 1 and 4 (DLL1 and DLL4), protein Wnt-3a (WNT3a), and epidermal growth factor receptor (EGFR) ligands transforming growth factor α (TGFα), together with the Wnt receptor frizzled 5 (FZD5). These signaling pathways maintain balance in activating and differentiating stem cells. Paneth cells have in their structure granules full of microbicidal proteins, including α-defensins, C-type lectins (CLEC), lysozyme and phospholipase A2, which are released into the intestinal lumen after the detection of the microbial signals [126,127,128].

Intestinal epithelial cells are permanently connected by structures of tightly semipermeable “apical junctional complexes” for the influx of ions and dissolved substances less than 600 Da and, at the same time, should stop the penetration of pathogens [129,130].

Tight junctions are made up of several transmembrane and cytosolic proteins, as follows: occludin, claudins, zonula occludens (ZO), tricellulin, cingulin, angulins and junctional adhesion molecules (JAM), which are interconnected in an intricate framework, acting on each other, as well as on the cytoskeleton. Cingulin and ZO are cytoskeletal binding proteins that work together with the peripheral membrane cytoplasmic proteins, occludin, claudins and JAM to create powerful cross-links and interrelate with the membrane cytoskeleton (composed of F-actin and myosin). TJ complexes are extremely dynamic, continuously transmit signals to other adjacent structures, quickly open and close the intestinal barrier, seal the paracellular pathway and conduct “gate and fence” functions. TJ proteins, along with the intracellular signaling proteins, are operating a multitude of cellular activities to modulate the integrity of the intestinal barrier [131,132].

Our scientific knowledge of TJs functions has been deepened by the identification of an important family of physiological regulatory proteins of TJs, with a unique recognized signaling mechanism. Tight junctions consist of the transmembrane proteins occludin and claudin and the cytoplasmic scaffolding proteins ZO-1, -2, and -3. The last are members of the MAGUK (membrane-associated guanylate kinase homologs) family with binding domains to adherens and TJ proteins in addition to the actin cytoskeleton, showing a great capability of interaction with diverse cellular proteins via lots of protein binding regions and important roles in regulating intestinal permeability. The ZO group are made of intracellular proteins interconnecting TJ transmembrane proteins to the actin cytoskeleton and making steadfast the TJ filaments, just like a scaffold network. The cells that do not have both ZO-1 and -2 cannot create TJs [133,134,135,136].

Zonulin is a mammalian analogue of the zonula occludens toxin (Zot), secreted by *Vibrio cholerae* and has been shown to be involved in the pathogenesis of many diseases. The family of zonulin are proteins connected architecturally and functionally to pre-haptoglobin (HP) 2 and its mature isoform HP2, being the first constituent discovered and expressed only in individuals carrying the HP2 allele, with a fundamental role in chronic inflammatory diseases or autoimmune, such as CD and T1DM, caused by the loss of zonulin-modulated small intestine epithelial barrier and contributing to innate intestinal immunity [137,138,139,140,141].

The stimuli involved in the release of zonulin are bacteria and gliadin. The whole framework for controlling the intestinal barrier permeability by zonulin is as follows: the gliadin or bacteria from the unbalanced microbiome will bind to the Chemokine (C-X-C Motif) Receptor 3 (CXCR3), and consequently, the MyD88-dependent zonulin will be released in the lumen of the small intestine; activity is further performed with the involvement of EGFR and protease-activated receptor 2 (PAR2); the transactivation of zonulin by EGFR through PAR2, in turn, activates phospholipase C (PLC), which stimulates a cascade of biological events that will lead to the increase of intracellular Ca, and the activation of protein kinase C alpha (PKCα)-dependent TJs disassembly. In fact, the activated PKCα increases the phosphorylation of ZO-1, ZO-2 and myosin 1C (MYO1C) in addition to the F-actin polymerization. As a side effect, ZO-1 will weaken the epithelial TJs and increase intestinal permeability. The raised intestinal permeability allows the paracellular fluxes of non-self-antigens to enter the lamina propria, which are processed by the immune system. Zonulin renders inactive by proteolytic degradation by trypsin IV.

In the case of subjects genetically predisposed to autoimmune diseases, changes in the structure and function of their intestinal microbiota caused by environmental factors will disrupt the function of the zonulin-dependent intestinal barrier (inadequate control of antigen flows), i.e., zonulin-dependent loss of the intestinal mucosal barrier, leading to “leaky gut” or the “permeable intestine” and a dysfunctional immune response of the mucosa, with implications for autoimmunity and chronic inflammatory diseases [134,139,142,143,144].

In another recent clinical study, Yonker et al. addressed the hypothesis that the passage of SARS-CoV-2 viral particles from the intestinal lumen into the systemic circulation could trigger fulminant cases of MIS-C. Persistence of the virus for weeks or even months after contact or initial infection with SARS-CoV-2 will result in the release of zonulin from intestinal epithelial cells and will weaken TJs, facilitating the passage of highly inflammatory viral structures into the systemic circulation. The authors extended the compassionate administration of larazotide to four patients aged 3 to 17 years (median, 7.5 years), diagnosed with MIS-C, following approvals from FDA and the Institutional Review Board (IRB), with parental and/or patient consent. All patients received larazotide at a dose of 10 mcg/kg orally four times a day for 21 days as an adjuvant medication to steroids, IVIG and anakinra. The study was performed in comparison with 22 other patients with MIS-C (with an average age of wight years) who did not receive larazotide treatment, used as a control group. Monitored parameters included the laboratory data represented by C-reactive protein (CRP), d-dimers, anti-SARS-CoV-2 Spike, -S1 and -S2 subunits antibodies, and an anti-RBD antibody, as well as the clinical symptoms. The four studied cases presented positive serologic evidence for prior SARS-CoV-2 infection and had detectable SARS-CoV-2 antigenemia at hospitalization. All four patients had important GI symptoms with significant multi-organ involvement, and two also showed cardiac damage. There was a significant decrease in the intensity and duration of GI symptoms in the larazotide group, and the time to discharge was slightly shorter than in the control group. It is noteworthy that the level of spike antigens cleared in one day, much faster in the larazotide-treated group, compared to 5.5 days in the control group.

The authors looked at four children with MIS-C who showed positive results when they were administered larazotide as adjuvant therapy, compared with 22 patients managed only with steroids and/or IVIG. The four children had a faster remission of GI symptoms and a shorter time to complete elimination of the spike antigen, demonstrating an amelioration in GI mucosal barrier activity, as well as a shorter time to hospital discharge, results suggesting that larazotide is a secure and useful as adjuvant therapy for MIS-C. The authors considered additional future double-blind, randomized, placebo-controlled studies to further investigate the effectiveness of this drug. The results showed that the only exclusive suppression of the immune system might not be the most favorable management for MIS-C, and new treatments, such as a zonulin agonist, which is aimed exactly at the origins of antigen “leak” into the systemic circulation, would be promising for MIS-C.

Even weeks or months after the disappearance of the clinical signs, patients with MIS-C showed a possible autoimmune signature left by the still present antigen concentrations, i.e., a continuous antigenemia, with yet unexplained consequences. AI disorders, abnormalities or immune impairments may not be noticeable for years and should be monitored. Therefore, the MIS-C patients should receive long-term surveillance for possible autoimmune outcomes and compared with those treated with larazotide. Thus, these and other results call attention to the antigenemia of SARS-CoV-2 as a possible biomarker, especially when coupled with high levels of zonulin, which could predict MIS-C and initiate early therapy. Only a follow-up and an in-depth look at circulating levels of antibodies-antigen over time can help elucidate the origins of the immune disorder. A certain increase in S1 or spike concentrations suggests that a false antigen mask may obscure the real values. Through these presented cases, the authors introspectively objectify MIS-C and focus on a possible new therapeutic pathway [145].

## 5. Discussions

The COVID-19 pandemic has not ended, and the number of children and adolescents infected with SARS-CoV-2 has increased dramatically worldwide in 2022, with the emergence of the highly transmissible Omicron variant. In the United States, a peak of 1,150,000 cases was reported in just one week. For example, between 12 and 19 May, more than 107,000 cases of COVID-19 were reported in children, 72% more than the previous week. Since the beginning of the pandemic, approximately 13.3 million children in the United States have tested positive for SARS-CoV-2, of which 5.4 million cases have been reported in 2022 [146].

For patients with MIS-C, the most recent case update from the Centers for Disease Control and Prevention in the US before 2 May 2022 was as follows: the total number of MIS-C patients was 8210, and the total number of MIS-C deaths was 68. About 50% of children with MIS-C were between 5 and 13 years old (mean age of 9 years), and 61% were male. Of the children diagnosed with SARS CoV-2, 98% had a positive test, and the difference between 2% had contact with someone diagnosed with COVID-19 [147].

A particularly important aspect is that during the pandemic in children who manifested life-threatening forms, i.e., MIS-C, GI symptoms were observed in over 80% of cases, compared to only 10–15% among adults infected with SARS-CoV-2. The difference in the late-onset of GI symptoms, together with the delayed onset of MIS-C in relation to the moment of infection or contact with a subject diagnosed with COVID, leads to the idea that the pathophysiological mechanisms in patients with MIS-C are different from that of adults with active COVID-19 [148,149,150].

At the level of the intestine, the defense against the aggressions of external environmental factors (microbes, viruses, RNA fractions, food components, toxins, etc.) begins by restricting the direct intimacy of these aggressors with the host cells and tissues. This protection is obtained through the intestinal barrier composed of mucus, locally secreted antimicrobial molecules, epithelial cells, and IgA secretory released by plasma cells in the lamina propria and the intestinal epithelium [151].

The second mode of defense is given by the innate immune system through its pattern recognition receptors (PRRs) in intestinal epithelial cells [Toll-like receptor-4 (TLR4) and lymphocyte antigen 96, known as Myeloid Differentiation factor 2 (MD-2)] that maintain a balance of tolerance to intestinal microbiota and food products in the homeostatic phase. The third strategy is the unique regulatory network of the mucosal immune system. Macrophages in the lamina propria of the intestinal mucosa play an important role both in maintaining intestinal homeostasis and in defending against foreign aggressors. Under homeostatic physiological conditions, the macrophages in the lamina propria attract microorganisms or foreign products from the intestinal lumen and release a small amount of pro-inflammatory as well as anti-inflammatory cytokines [152].

The innate immune response to the external aggressor agent is triggered by the recognition of pathogen-associated molecular patterns (PAMPs) by pathogen-recognition receptors (PRRs), namely Toll-like receptors (TLRs) on the surface of cells or in the endosomes of intestinal epithelial cells, macrophages, dendrites, B and T cells, as well as stromal cells. Cytoplasmic PRRs include the RNA helicase family and the nucleotide-binding and oligomerization domain (NOD)-like receptor (NLR) family [153].

Recent publications have suggested that disruption of the microbiota and impairments in intestinal barrier function triggers local inflammation by activating the immune system and causing severe COVID-19 infections on the gut–lung–brain axis in adults [154,155] and likely in children diagnosed with MIS-C. GI dysfunction in MIS-C is under ongoing investigation [156,157].

**Figure 1:**
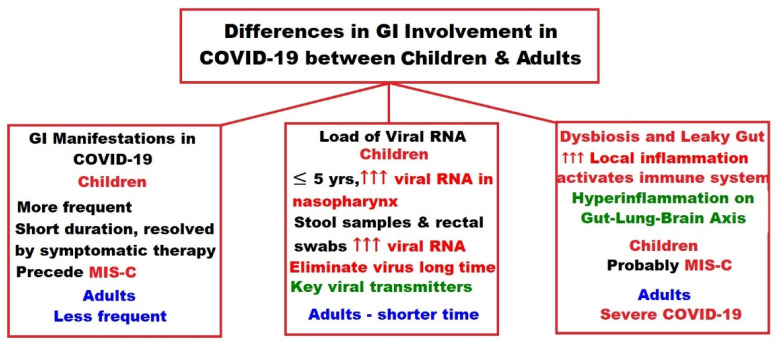
Comparative representation of children—adults of gastrointestinal involvement in COVID-19. Legend: ↑↑↑ = “Very high”.

The differences between children and adults in connection with the previously mentioned gastrointestinal implications of COVID-19 are depicted in Figure 1.

A very important role in regulating the permeability and barrier function of the intestinal epithelium depends on intercellular TJs. Several proteins have been discovered that play a key role in paracellular permeability. The most studied of this junctional protein complex is ZO-1, a protein whose C-terminus is functionally bound to the cytoskeleton of the cell, while the N-terminus binds to the occludin, a TJ protein. Zonulin, already known to be an important modulator of TJs and intestinal barrier functions, is secreted by intestinal epithelial cells when stimulated by dietary or local infectious factors. Infectious aggression can lead to the destruction of the intestinal barrier by apoptosis of intestinal epithelial cells, which will generate a strong proinflammatory environment with the differentiation of autoreactive Th17 and other T-helper cells [136,158,159].

The researcher who discovered zonulin highlighted an extremely important function of the GI tract, namely the regulation of macromolecule traffic between the environment and the host through an intestinal epithelial barrier mechanism. He proposed a third decisive factor acting on the path of autoimmunity, namely the intestinal permeability, along with genetic predisposition and environmental factors. Basically, the balance between tolerance and immunity to non-self-antigens is controlled by the GI tract through the intestinal epithelial barrier with its intercellular TJs, along with the associated lymphoid tissue and the neuroendocrine network. Based on the research in recent decades, a new hypothesis on the trajectory of autoimmunity has been rethought and reshaped to explain the multitude of autoimmune diseases occurring more and more frequently in both the elderly and young. Thus, in genetically predisposed subjects, a disturbance of the fine-regulated mechanisms of the zonulin-controlled pathway could induce autoimmune diseases, intestinal or extraintestinal, such as CD, but also inflammatory or even neoplastic [138].

**Figure 2:**
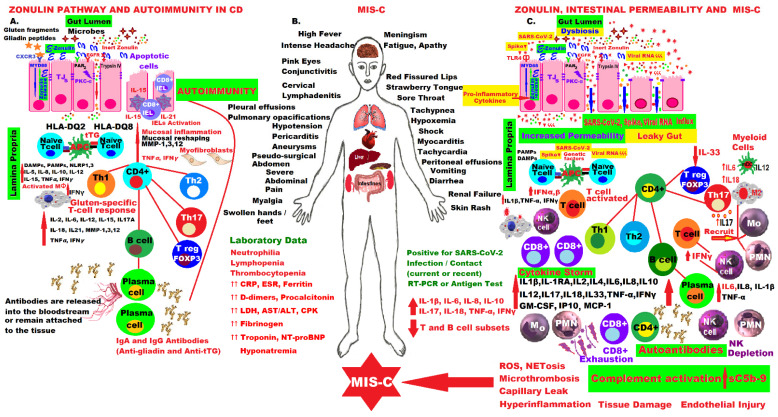
Comparative molecular mechanisms in the pathogenesis of CD and MIS-C by activating the zonulin pathway, increasing intestinal permeability, hyperinflammation and immune dysregulation (↑ = Increased; ↑↑ = High). (**A**). The undigested fragments of gluten peptides in the intestinal lamina propria are attacked and deamidated by tTG, then taken up and presented to HLA-DQ2 and DQ8 molecules by DC and via T-helper cells will initiate an adaptive immune response. The gliadin peptides bound to the TLR2 receptors will influence the increase in cytokine production through MYD88, the key protein involved in the release of zonulin after gluten ingestion. The transactivation of zonulin by EGFR through PAR2 stimulates a cascade of biological events that finally will lead to the activation of PKCα-dependent TJs disassembly. Zonulin renders inactive by proteolytic degradation by trypsin IV. Practically, zonulin will weaken the epithelial TJs and increase intestinal permeability. A gluten-specific T-cell response will initiate a cascade of events, followed by the activation of B and plasma B cells, the release of IgA and IgG antibodies (anti-gliadin and anti-tTG), the mucosal inflammation and reshaping, and the initiation of the autoimmune processes. (**B**). Clinical picture and dramatically altered biological data in MIS-C. (**C**). Following the ingestion of the SARS-CoV-2 virus and its presence in the lumen of the small intestine, its spike proteins and RNA fractions, in conjunction with the intestinal dysbiosis, bind to TLR receptors and will influence the increase in pro-inflammatory cytokines production through MYD88, and the release of zonulin, exactly as described above in section A. A zonulin-dependent loss of the intestinal mucosal barrier will lead to a “leaky gut” with subsequent trafficking of SARS-CoV-2 antigens into the bloodstream and a dysfunctional immune response, i.e., immune hyperactivation, massive cytokine production (cytokine storm), huge release of antibodies, complement activation, microthrombosis, severe systemic inflammation, capillary leak, endothelial and tissue damage, and finally multiple organ dysfunction (MIS-C). [Figure 2 was imagined and drawn by L.M.A. using Microsoft Paint 3D (3D Library—Biology: human heart and brain) for Windows 10 and using completely free picture material (human lungs, kidney, intestines, and capillaries clip arts) from SeekPNG.com (accessed on 4 June 2022), for which we are very grateful].

Figure 2 illustrates the molecular mechanisms involved in zonulin pathway activation and the pathophysiology of CD compared to the molecular aspects of fulminant forms of MIS-C that occurred in children a few weeks after infection or contact with the SARS-CoV-2 virus. The patient has alarming symptoms that must be recognized immediately and usually require emergency hospitalization in intensive care units, as they are life-threatening. Mastery of these intrinsic molecular mechanisms and the hypothesis of activation of the zonulin pathway and loss of the intestinal mucosal barrier led to the proposal of an adjuvant drug for MIS-C in this pandemic.

It has been shown that gluten, bacteria and other microorganisms can stimulate the release of zonulin. This can be used as a biomarker to highlight the change in paracellular permeability of the small intestine, as it leads to the disassembly of TJs through phosphorylation reactions, followed by polymerization, the redistribution of actin filaments, and the displacement of the ZO-1 protein. In this context, it has been found that zonulin has very high serum levels in CD and T1DM and correlates well with the density of intestinal microorganisms. CD, intestinal permeability and diabetes incidence could be modified by diet, but it should be mentioned that the molecular biomarkers of intestinal permeability are hard to interpret [160].

People with CD, mainly untreated patients, may be at higher risk for infections such as COVID-19 [98,161].

Especially in developed countries, the use of new diagnostic criteria with specific antibodies have shortened the diagnostic time [15,162].

Most recent published studies focus on childhood infections, which could lead to alteration of the intestinal microbiota following the administration of antibiotics [163,164,165,166].

Viruses and other microbial agents have a direct influence on the immune reactions of the lining of the small intestine and may increase the sensitivity to the action of gluten. The essential enzyme in triggering CD, the tTG, is released more easily during viral infections. Side effects of infectious aggression are evidenced by the involvement of the pathogen-associated molecular patterns (PAMPs) and the damage-associated molecular patterns (DAMPs) in activating the innate immune system by stimulating T lymphocytes that open the inflammatory window to CD pathogenesis [167,168,169].

During acute viral infections, regulatory T lymphocytes (Tregs), a subpopulation of T cells (FoxP3^+^ CD4^+^CD25^+^), play an essential role in controlling inflammation and preventing autoimmunity and tissue complications by regulating immune system homeostasis. Human FOXP3^+^ cluster of differentiation (CD)25^+^CD4^+^ Tregs are a type of T cells that express CD4, CD25 and FOXP3, which are critical for maintaining immune homeostasis [170].

Gluten consumption acts on the small intestine by triggering a cascade of inflammatory events that are secondary to innate and adaptive immunity responses. Many studies have shown the important role of IL-6 in triggering CD. IL-6, as a pleiotropic cytokine produced by different cell types, has a dual activity through its pro- and anti-inflammatory effects and during pro-inflammatory activity, it increases the synthesis of acute-phase proteins and can induce an uncontrolled inflammatory process and even the onset of CD [171,172,173].

In the attempt to discover the relationship between IL-6, SARS-CoV-2 infection and CD, it can be said that, after the onset of infection, the release of IL-6 as a proinflammatory marker is beneficial in controlling viral infection and bacterial complications. Disturbance of IL-6 production is associated with the onset, progression and severity of respiratory, cardiovascular, and digestive manifestations and even mortality in patients with COVID-19 [174,175,176].

Increased serum levels of IL-6 were observed in patients with CD during non-GFDs, returning to normal values only after one year of GFD. Elevated levels of IL-6 in CD boost proinflammatory activity and support the differentiation of T-helper 17 (Th17) lymphocytes, with a negative impact on the intestinal mucosa. Increased production of proinflammatory cytokines, including IL-6, is linked to autoimmune disorders and other key factors in the cytokine storm in patients with severe COVID-19 [171,177,178].

In the recently published study by Asri et al., it is suggested that high levels of IL-6 may predispose non-dietary CD patients to severe complications if infected with SARS-CoV-2. In this case, improved expression of CD4, CD25 and FOXP3 as anti-inflammatory markers may be helpful in reducing the serious adverse events of SARS-CoV-2 infection, as demonstrated in the final conclusions of this clinical trial with the control group [98,179].

CD as an immune-mediated disease is associated with an increased risk of infections, including COVID-19, due to nutritional, vitamin and mineral deficiencies, especially for patients who do not comply with GFD. Multidisciplinary healthcare projects are needed to improve the QOL of these children [37,180,181,182].

Hadi et al. performed a retrospective cohort analysis of a total of 341,499 patients over the age of 16 identified in the TriNETX multicenter research network over 30 to 60 days post-SARS-CoV-2 infection. The authors found only 930 (0.27%) patients with CD and 340,569 (99.73%) without CD; the hospitalization percentage for CD patients was 8.71%, and mortality rates in CD patients were 1.29%, compared to 1.40% in non-CD patients, and no disparity in the demand for hospitalization, needs critical care or acute kidney injury in CD patients compared to patients without CD [183].

In a very recent study, Greco et al. evaluated a group of 191 CD patients ≥18 years of age with HLA haplotype DQ2 and/or DQ8 through a questionnaire on actual clinical symptoms, psychological effects, the difficulties of maintaining a GFD during the COVID-19 pandemic, and the possible SARS-CoV-2 infection. Results showed that patients had no difficulty in preserving a GFD; only 5.8% of patients tested positive for SARS-CoV-2 infection, but the clinical symptoms were mild and did not require hospitalization or intensive care. Based on the results, the authors’ new assumption that the HLA haplotype DQ2 and/or DQ8 would play a protective role in patients with CD against viral infections, including SARS-CoV-2, is quite interesting and should be further investigated [184].

In another study published by Samasca et al., it was found that patients with CD did not have a higher risk of SARS-CoV-2 infection but increased psychological distress. This COVID-19 pandemic has revealed shortcomings in the education of patients with CD, but especially those associated with T1DM or IBD, for GFD compliance. Adherence of CD patients to GFD during the COVID-19 pandemic improved their QOL and prevented unwanted complications [185].

COVID vaccination has opened a new chapter in this pandemic, and we still have much to do in all areas, including medicine, to deepen our understanding of all mechanisms, to better care for our patients and protect us from future waves [186].

Some authors suggest that MIS-C should be reinterpreted as a special macrophage activation syndrome, and long-term protection against SARS-CoV-2 infection can only be provided by the vaccine, but we do not yet have sufficient data [187].

Vaccination provides the best solution for controlling the COVID-19 pandemic, and patients with chronic inflammatory and autoimmune diseases, including CD, need to be convinced of the necessity, safety and efficacy of vaccines, even if they have been produced in a very short time, generating high levels of risk perception, different attitudes, significant debates on acceptance and great hesitation worldwide [188,189,190,191].

Vaccination in children with CD is one of the best tools to slow down or stop the spread of the virus. Daily physical activity and vaccination are excellent methods for maintaining digestive and general health, including the immune system. Patients with CD can receive any of the vaccines available on the market that are safe and effective in preventing COVID-19. There is no increased risk of side effects in patients with CD compared to the general population, as none of the current vaccines contains gluten or prolamins.

**Figure 3:**
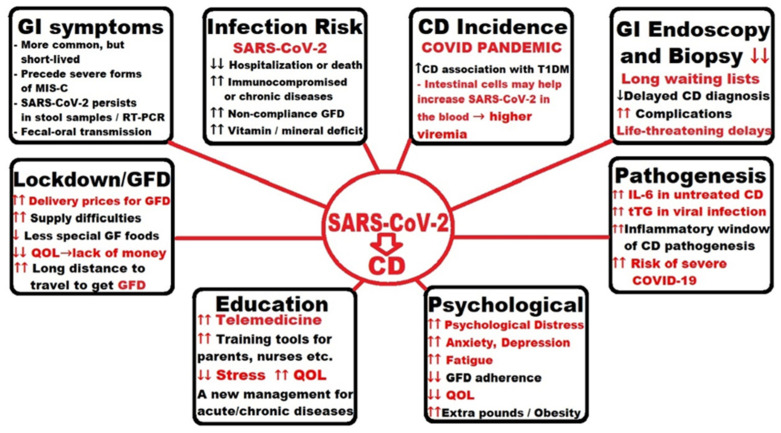
Consequences of the COVID-19 pandemic in children with CD (↑ = Increased; ↑↑ = High; ↓ = Decreased, ↓↓= Lower).

The complex impact of SARS-CoV-2 infection in children diagnosed with CD addressed in this review is summarized and illustrated in Figure 3.

## 6. Conclusions

This review highlighted that the risk of infection and death due to COVID-19 was not higher in CD patients than in the general population.

The highest risks of contracting the infection were observed in immunocompromised patients and in those with nutritional deficiencies, especially in patients with CD who did not comply with GFD.

Incidence of CD diagnosis has increased, but especially in association with T1DM, although the number of intestinal biopsies has decreased. Long waiting lists for GI endoscopies have increased complications and caused life-threatening delays, especially in young children.

COVID-19 pandemic caused shortcomings in GFD adherence due to high delivery prices, supply difficulties, long travel distances to obtain GFD, reduced family income, and decreased QOL through the lockdown.

For patients with CD, the pandemic caused psychological distress, insomnia, irritability, anxiety, chronic fatigue, depression, decreased quality of life, low compliance with GFD and metabolic complications such as obesity and diabetes.

Patients with CD can receive any of the vaccines available on the market that are safe and effective in preventing COVID-19, as none of the current vaccines contains gluten or prolamins.

Introspection into the molecular pathophysiological mechanisms of SARS-CoV-2 infection and profound similarity in the disruption of mucosal integrity in CD led to the proposal of a CD-inspired drug for MIS-C, a zonulin antagonist.

As the pandemic is not over and there are still cases of MIS-C, further studies are needed to pave the way for understanding the pathophysiological mechanisms of this fulminant disease.

An ongoing challenge is to imagine new delivery platforms and new molecules as immunotherapies for resolving immune-related diseases and for balancing the response of the GI immune system as a multi-field sovereign system.

Zonulin is widely studied in immunoengineering as an adjunct to improving the absorption of new oral drugs and vaccines.

In the near future, scientists should develop innovative approaches to combat high rates of autoimmune diseases.

## Data Availability

Literature supporting reported results can be found in the list of references.

## Abbreviations

Angiotensin-converting enzyme 2	ACE-2
α-amylase/trypsin	ATI
Anti-endomysium antibodies IgA	anti-EMA-IgA
Antigen-presenting cell	APC
Anti-tissue transglutaminase IgA antibodies	anti-tTG IgA
Aspartate transaminase/Alanine transaminase	AST/ALT
Autoimmune	AI
Before Common Era	BCE
Casein kinase 1, alpha 1	CSNK1A1
Casein kinase 2, alpha 1	CSNK2A1
Casein kinase 1, epsilon 1	CSNK1E1
Celiac disease	CD
Chemokine (C-C motif) ligand 3	CCL3
Chemokine (C-C motif) ligand 4	CCL4
Chemokine (C-C motif) ligand 20	CCL20
Chemokine (C-C motif) ligand 28	CCL28
Chemokine (C-X-C Motif) Receptor 3	CXCR3
Complement	C
Coronavirus Disease 2019	COVID-19
Creatine phosphokinase	CPK
Crypt base columnar	CBC
C-reactive protein	CRP
C-type lectins	CLEC
CUB domain-containing protein 1	CDCP1
Damage-associated molecular pattern	DAMP
Deamidated gliadin peptide	DGP
Deamidated gliadin peptide antibodies	DGP-AGA
Delta-like canonical Notch ligand 1	DLL1
Delta-like canonical Notch ligand 4	DLL4
Dendritic cell	DC
Epidermal growth factor	EGF
Epidermal growth factor receptor	EGFR
Erythrocyte sedimentation rate	ESR
European Society for Paediatric Gastroenterology Hepatology and Nutrition	ESPGHAN
Food and Drug Administration	FDA
Forkhead box P3—also known as Scurfin	FOXP3
Gastrointestinal	GI
Gluten-free	GF
Gluten-free diet	GFD
Pre-haptoglobin	HP
High-mobility group protein 1	HMGB1
Human leukocyte antigen	HLA
IgA tissue transglutaminase	tTGA
IgA anti-endomysium antibodies	EMA-IgA
IgA anti-intestinal transglutaminase 2 antibodies	TGA-IgA
IgG against deamidate gliadin peptide	DGP-IgG
Immunoglobulin type A	IgA
Immunoglobulin type G	IgG
Immunoglobulin type M	IgM
Inflammatory bowel disease	IBD
Institutional Review Board	IRB
Interferon-γ	IFN-γ
Interleukin	IL-
Intraepithelial CD8+ lymphocytes	IEL
Intravenous immune globulins	IVIG
Junctional adhesion molecules	JAM
Kawasaki disease	KD
Lactate dehydrogenase	LDH or LD
Matrix metalloproteinase-1	MMP-1
Membrane-associated guanylate kinase homologs	MAGUK
Dual specificity mitogen-activated protein kinase kinase 2	MAP2K2
Microbial associated molecular pattern	MAMPs
Middle East Respiratory Syndrome	MERS
Monocyte	Mo
Monocyte chemoattractant protein-1	MCP-1
Multisystem inflammatory syndrome in children	MIS-C
Myeloid differentiation primary response 88	MYD88
Myosin 1C	MYO1C
Natural killer	NK
Neutrophil extracellular traps	NETs
NET activation and release	NETosis
(NOD)-like receptor	NLR
N-terminal prohormone of brain natriuretic peptide	NT-proBNP
Pathogen-associated molecular pattern	PAMP
Pattern recognition receptors	PRRs
Pediatric inflammatory multisystem syndrome	PIMS
Phospholipase C	PLC
Polymorphonuclear leukocyte	PMN
Protease-activated receptor 2	PAR2
Protein kinase C alpha	PKCα
Protein Wnt-3a	WNT3a
Quality of life	QOL
Reactive oxygen species	ROS
Real-time polymerase chain reaction	Real-time PCR or RT-PCR
Receptor Binding Domain	RBD
Regulatory T lymphocytes	Tregs
Ribonucleic acid	RNA
Secretory IgA	SIgA
Severe Acute Respiratory Syndrome	SARS
Severe acute respiratory syndrome coronavirus 2	SARS-CoV-2
Soluble C5b-9	sC5b-9
Systemic inflammatory syndrome in COVID19	SISCoV
Systemic juvenile idiopathic arthritis	sJIA
Temporally associated with SARS-CoV-2 infection	PIMS-TS
T-helper 1 or T helper type 1	Th1
T-helper 2 or T helper type 2	Th2
T-helper 17 or T helper type 17	Th17
Tight junctions	TJs
Tissue transglutaminase or tissue transglutaminase autoantigen	tTG/tTG2
Toll-like receptor 2	TLR2
Toll-like receptor 4	TLR 4
Transforming growth factor alpha	TGF-α
Transglutaminase 2	TG2
Transmembrane serine protease 2	TMPRSS2
Tumor necrosis factor alpha	TNF-α
Type-1 diabetes mellitus	T1DM
Upper limit of normal	ULN
Wnt receptor frizzled 5	FZD5
World Health Organization	WHO
Wnt Family Member 3	WNT3a
Zonula occludens	ZO
Zonula occludens-1	ZO-1
Zonula occludens toxin	Zot
**↑**	Increased
**↓**	Decreased

## References

[1] Mallah S.I., Ghorab O.K., Al-Salmi S., Abdellatif O.S., Tharmaratnam T., Iskandar M.A., Sefen J., Sidhu P., Atallah B., El-Lababidi R. (2021). COVID-19: Breaking down a global health crisis. Ann. Clin. Microbiol. Antimicrob..

[2] Zhen J., Stefanolo J.P., Temprano M.P., Tedesco S., Seiler C., Caminero A.F., de-Madaria E., Huguet M.M., Vivas S., Niveloni S.I. (2021). The Risk of Contracting COVID-19 Is Not Increased in Patients With Celiac Disease. Clin. Gastroenterol. Hepatol..

[3] WHO Naming the Coronavirus Disease (COVID-19) and the Virus That Causes It. https://www.who.int/emergencies/diseases/novel-coronavirus-2019/technical-guidance/naming-the-coronavirus-disease-(covid-2019)-and-the-virus-that-causes-it.

[4] Domingo J.L. (2021). What we know and what we need to know about the origin of SARS-CoV-2. Environ. Res..

[5] WHO Coronavirus (COVID-19) Dashboard. https://covid19.who.int.

[6] Fasano A. (2020). All disease begins in the (leaky) gut: Role of zonulin-mediated gut permeability in the pathogenesis of some chronic inflammatory diseases. F1000Research.

[7] Lynch S.V., Pedersen O.=. (2016). The Human Intestinal Microbiome in Health and Disease. N. Engl. J. Med..

[8] Proctor L. (2018). The NIH Human Microbiome Project: Catalyst for an Emerging Field in Biomedical Research. https://www.genome.gov/Pages/About/NACHGR/February2018AgendaDocuments/HMP_talk_Feb_Council_final_020618.pdf.

[9] The Editors of Encyclopaedia Britannica Small Intestine. Encyclopedia Britannica. https://www.britannica.com/science/small-intestine.

[10] Children’s Hospital of Pittsburgh Difference between Small and Large Intestine. https://www.chp.edu/our-services/transplant/intestine/education/about-small-large-intestines.

[11] Zuo L., Kuo W.T., Turner J.R. (2020). Tight Junctions as Targets and Effectors of Mucosal Immune Homeostasis. Cell. Mol. Gastroenterol. Hepatol..

[12] Sahin Y. (2021). Celiac disease in children: A review of the literature. World J. Clin. Pediatr..

[13] Husby S., Koletzko S., Korponay-Szabó I., Kurppa K., Mearin M.L., Ribes-Koninckx C., Shamir R., Troncone R., Auricchio R., Castillejo G. (2020). European society Paediatric gastroenterology, hepatology and nutrition guidelines for diagnosing coeliac disease 2020. J. Pediatr. Gastroenterol. Nutr..

[14] Singh P., Arora A., Strand T.A., Leffler D.A., Catassi C., Green P.H., Kelly C.P., Ahuja V., Makharia G.K. (2018). Global Prevalence of Celiac Disease: Systematic Review and Meta-analysis. Clin. Gastroenterol. Hepatol..

[15] Lindfors K., Ciacci C., Kurppa K., Lundin K.E.A., Makharia G.K., Mearin M.L., Murray J.A., Verdu E.F., Kaukinen K. (2019). Coeliac disease. Nat. Rev. Dis. Prim..

[16] Laurikka P., Nurminen S., Kivelä L., Kurppa K. (2018). Extraintestinal Manifestations of Celiac Disease: Early Detection for Better Long-Term Outcomes. Nutrients.

[17] Lebwohl B., Murray J.A., Verdú E.F., Crowe S.E., Dennis M., Fasano A., Green P.H., Guandalini S., Khosla C. (2016). Gluten Introduction, Breastfeeding, and Celiac Disease: Back to the Drawing Board. Am. J. Gastroenterol..

[18] Jiang L.L., Zhang B.L., Liu Y.S. (2009). Is adult celiac disease really uncommon in Chinese?. J. Zhejiang Univ. Sci. B.

[19] Wang X.Q., Liu W., Xu C.D., Mei H., Gao Y., Peng H.M., Yuan L., Xu J.J. (2011). Celiac disease in children with diarrhea in 4 cities in China. J. Pediatr. Gastroenterol. Nutr..

[20] Wang M., Kong W.J., Feng Y., Lu J.J., Hui W.J., Liu W.D., Li Z.Q., Shi T., Cui M., Sun Z.Z. (2022). Epidemiological, clinical, and histological presentation of celiac disease in Northwest China. World J. Gastroenterol..

[21] Zhou W.Y., Liu X.Y., Wang M.M., Liang L.P., Liu L., Zheng K., Silvester J.A., Ma W.J., Wu W., Ji G.Y. (2021). Prevalence of celiac disease in China: Meta-analysis and serological survey in high-risk populations. J. Dig. Dis..

[22] Tarar Z.I., Zafar M.U., Farooq U., Basar O., Tahan V., Daglilar E. (2021). The Progression of Celiac Disease, Diagnostic Modalities, and Treatment Options. J. Investig. Med. High Impact Case Rep..

[23] Abenavoli L., Dastoli S., Bennardo L., Boccuto L., Passante M., Silvestri M., Proietti I., Potenza C., Luzza F., Nisticò S.P. (2019). The Skin in Celiac Disease Patients: The Other Side of the Coin. Medicina.

[24] Hadithi M., von Blomberg B.M., Crusius J.B., Bloemena E., Kostense P.J., Meijer J.W., Mulder C.J., Stehouwer C.D., Peña A.S. (2007). Accuracy of serologic tests and HLA-DQ typing for diagnosing celiac disease. Ann. Intern. Med..

[25] Hoffmanová I., Sánchez D., Szczepanková A., Tlaskalová-Hogenová H. (2019). The Pros and Cons of Using Oat in a Gluten-Free Diet for Celiac Patients. Nutrients.

[26] Caio G., Volta U., Sapone A., Leffler D.A., De Giorgio R., Catassi C., Fasano A. (2019). Celiac disease: A comprehensive current review. BMC Med..

[27] Maglio M., Troncone R. (2020). Intestinal Anti-tissue Transglutaminase2 Autoantibodies: Pathogenic and Clinical Implications for Celiac Disease. Front. Nutr..

[28] Lionetti E., Castellaneta S., Francavilla R., Pulvirenti A., Catassi C. (2017). SIGENP Working Group of Weaning and CD Risk. Mode of Delivery and Risk of Celiac Disease: Risk of Celiac Disease and Age at Gluten Introduction Cohort Study. J. Pediatr..

[29] Koletzko S., Lee H.S., Beyerlein A., Aronsson C.A., Hummel M., Liu E., Simell V., Kurppa K., Lernmark Å., Hagopian W. (2018). TEDDY Study Group. Cesarean Section on the Risk of Celiac Disease in the Offspring: The Teddy Study. J. Pediatr. Gastroenterol. Nutr..

[30] Zanoni G., Navone R., Lunardi C., Tridente G., Bason C., Sivori S., Beri R., Dolcino M., Valletta E., Corrocher R. (2006). In celiac disease, a subset of autoantibodies against transglutaminase binds toll-like receptor 4 and induces activation of monocytes. PLoS Med..

[31] Silvester J.A., Leffler D.A. (2017). Is Autoimmunity Infectious? The Effect of Gastrointestinal Viral Infections and Vaccination on Risk of Celiac Disease Autoimmunity. Clin. Gastroenterol. Hepatol..

[32] Hemming-Harlo M., Lähdeaho M.L., Mäki M., Vesikari T. (2019). Rotavirus vaccination does not increase type 1 diabetes and may decrease celiac disease in children and adolescents. Pediatr. Infect. Dis. J..

[33] Bouziat R., Hinterleitner R., Brown J.J., Stencel-Baerenwald J.E., Ikizler M., Mayassi T., Meisel M., Kim S.M., Discepolo V., Pruijssers A.J. (2017). Reovirus infection triggers inflammatory responses to dietary antigens and development of celiac disease. Science.

[34] Thomas K.E., Sapone A., Fasano A., Vogel S.N. (2006). Gliadin stimulation of murine macrophage inflammatory gene expression and intestinal permeability are MyD88-dependent: Role of the innate immune response in Celiac disease. J. Immunol..

[35] Neil J.A., Cadwell K. (2018). The Intestinal Virome and Immunity. J. Immunol..

[36] Sánchez D., Hoffmanová I., Szczepanková A., Hábová V., Tlaskalová-Hogenová H. (2021). Contribution of Infectious Agents to the Development of Celiac Disease. Microorganisms.

[37] Simons M., Scott-Sheldon L.A.J., Risech-Neyman Y., Moss S.F., Ludvigsson J.F., Green P.H.R. (2018). Celiac disease and increased risk of pneumococcal infection: A systematic review and meta-analysis. Am. J. Med..

[38] Canova C., Ludvigsson J., Baldo V., Barbiellini Amidei C., Zanier L., Zingone F. (2019). Risk of bacterial pneumonia and pneumococcal infection in youths with celiac disease-A population-based study. Dig. Liver Dis..

[39] Mårild K., Kahrs C.R., Tapia G., Stene L.C., Størdal K. (2015). Infections and risk of celiac disease in childhood: A prospective nationwide cohort study. Am. J. Gastroenterol..

[40] Van De Kamer J.H., Weijers H.A., Dicke W.K. (1953). Coeliac disease. IV. An investigation into the injurious constituents of wheat in connection with their action on patients with coeliac disease. Acta Paediatr..

[41] Auricchio S., De Ritis G., De Vincenzi M., Silano V. (1985). Toxicity mechanisms of wheat and other cereals in celiac disease and related enteropathies. J. Pediatr. Gastroenterol. Nutr..

[42] De Re V., Caggiari L., Tabuso M., Cannizzaro R. (2013). The versatile role of gliadin peptides in celiac disease. Clin. Biochem..

[43] De Re V., Magris R., Cannizzaro R. (2017). New Insights into the Pathogenesis of Celiac Disease. Front. Med..

[44] Biesiekierski J.R. (2017). What is gluten?. J. Gastroenterol. Hepatol..

[45] Sharma N., Bhatia S., Chunduri V., Kaur S., Sharma S., Kapoor P., Kumari A., Garg M. (2020). Pathogenesis of Celiac Disease and Other Gluten Related Disorders in Wheat and Strategies for Mitigating Them. Front. Nutr..

[46] Silano M., Vincentini O., De Vincenzi M. (2009). Toxic, immunostimulatory and antagonist gluten peptides in celiac disease. Curr. Med. Chem..

[47] Valenti S., Corica D., Ricciardi L., Romano C. (2017). Gluten-related disorders: Certainties, questions and doubts. Ann. Med..

[48] Cebolla Á., Moreno M.L., Coto L., Sousa C. (2018). Gluten Immunogenic Peptides as Standard for the Evaluation of Potential Harmful Prolamin Content in Food and Human Specimen. Nutrients.

[49] Clemente M.G., De Virgiliis S., Kang J.S., Macatagney R., Musu M.P., Di Pierro M.R., Drago S., Congia M., Fasano A. (2003). Early effects of gliadin on enterocyte intracellular signalling involved in intestinal barrier function. Gut.

[50] Moreno M.L., Cebolla Á., Muñoz-Suano A., Carrillo-Carrion C., Comino I., Pizarro Á., León F., Rodríguez-Herrera A., Sousa C. (2017). Detection of gluten immunogenic peptides in the urine of patients with coeliac disease reveals transgressions in the gluten-free diet and incomplete mucosal healing. Gut.

[51] Palanski B.A., Weng N., Zhang L., Hilmer A.J., Fall L.A., Swaminathan K., Jabri B., Sousa C., Fernandez-Becker N.Q., Khosla C. (2022). An efficient urine peptidomics workflow identifies chemically defined dietary gluten peptides from patients with celiac disease. Nat. Commun..

[52] Dunne M.R., Byrne G., Chirdo F.G., Feighery C. (2020). Coeliac Disease Pathogenesis: The Uncertainties of a Well-Known Immune Mediated Disorder. Front. Immunol..

[53] Zevallos V.F., Raker V., Tenzer S., Jimenez-Calvente C., Ashfaq-Khan M., Russel N., Pickert G., Schild H., Steinbrink K., Schuppan D. (2017). Nutritional wheat amylase-trypsin inhibitors promote intestinal inflammation via activation of myeloid cells. Gastroenterology.

[54] Schumann M., Siegmund B., Schulzke J.D., Fromm M. (2017). Celiac disease: Role of the epithelial barrier. Cell. Mol. Gastroenterol. Hepatol..

[55] Patel N., Robert M.E. (2022). Frontiers in Celiac Disease: Where Autoimmunity and Environment Meet. Am. J. Surg. Pathol..

[56] Kupfer S.S., Jabri B. (2012). Pathophysiology of celiac disease. Gastrointest. Endosc. Clin. N. Am..

[57] Parzanese I., Qehajaj D., Patrinicola F., Aralica M., Chiriva-Internati M., Stifter S., Elli L., Grizzi F. (2017). Celiac disease: From pathophysiology to treatment. World J. Gastrointest. Pathophysiol..

[58] Stamnaes J., Sollid L.M. (2015). Celiac disease: Autoimmunity in response to food antigen. Semin. Immunol..

[59] Palová-Jelínková L., Dáňová K., Drašarová H., Dvořák M., Funda D.P., Fundová P., Fundová P., Kotrbová-Kozak A., Černá M., Kamanová J. (2013). Pepsin Digest of Wheat Gliadin Fraction Increases Production of IL-1β via TLR4/MyD88/TRIF/MAPK/NF-κB Signaling Pathway and an NLRP3 Inflammasome Activation. PLoS ONE.

[60] Araya R.E., Gomez Castro M.F., Carasi P., McCarville J.L., Jury J., Mowat A.M., Verdu E.F., Chirdo F.G. (2016). Mechanisms of innate immune activation by gluten peptide p31-43 in mice. Am. J. Physiol. Liver Physiol..

[61] Tang D., Kang R., Berghe T.V., Vandenabeele P., Kroemer G. (2019). The molecular machinery of regulated cell death. Cell Res..

[62] Patankar J.V., Becker C. (2020). Cell death in the gut epithelium and implications for chronic inflammation. Nat. Rev. Gastroenterol. Hepatol..

[63] Perez F., Ruera C.N., Miculan E., Carasi P., Chirdo F.G. (2021). Programmed Cell Death in the Small Intestine: Implications for the Pathogenesis of Celiac Disease. Int. J. Mol. Sci..

[64] Kuja-Halkola R., Lebwohl B., Halfvarson J., Wijmenga C., Magnusson P.K., Ludvigsson J.F. (2016). Heritability of non-HLA genetics in coeliac disease: A population-based study in 107000 twins. Gut.

[65] Dubois P.C., Trynka G., Franke L., Hunt K.A., Romanos J., Curtotti A., Zhernakova A., Heap G.A., Adány R., Aromaa A. (2010). Multiple common variants for celiac disease influencing immune gene expression. Nat. Genet..

[66] Itzlinger A., Branchi F., Elli L., Schumann M. (2018). Gluten-Free Diet in Celiac Disease-Forever and for All?. Nutrients.

[67] Crehuá-Gaudiza E., Barrés Fernández A., Jovaní Casano C., Latorre Tejerina M., Largo Blanco E.M., Moreno Ruiz M.A., Berghezan Suárez A., García-Peris M., Gil Piquer R., Coret Sinisterra A. (2021). Diagnóstico de enfermedad celiaca en la práctica clínica: Presente y futuro. Diagnosis of celiac disease in clinical practice: Present and future. An. Pediatría.

[68] Parisi P., Pietropaoli N., Ferretti A., Nenna R., Mastrogiorgio G., Del Pozzo M., Principessa L., Bonamico M., Villa M.P. (2015). Role of the gluten-free diet on neurological-EEG findings and sleep disordered breathing in children with celiac disease. Seizure.

[69] Ben Houmich T., Admou B. (2021). Celiac disease: Understandings in diagnostic, nutritional, and medicinal aspects. Int. J. Immunopathol. Pharmacol..

[70] Sabino L., Marinot S., Falsaperla R., Pisani F., Massimino C., Pavone P. (2020). Celiac disease and headache in children: A narrative state of the art. Acta Biomed..

[71] Bao F., Green P.H., Bhagat G. (2012). An update on celiac disease histopathology and the road ahead. Arch. Pathol. Lab. Med..

[72] Volta U., Granito A., Parisi C., Fabbri A., Fiorini E., Piscaglia M., Tovoli F., Grasso V., Muratori P., Pappas G. (2010). Deamidated gliadin peptide antibodies as a routine test for celiac disease: A prospective analysis. J. Clin. Gastroenterol..

[73] Maheshwari A., He Z., Weidner M.N., Lin P., Bober R., Del Rosario F.J. (2021). Influence of Age and Type 1 Diabetes Mellitus on Serological Test for Celiac Disease in Children. Pediatr. Gastroenterol. Hepatol. Nutr..

[74] Kulkarni A., Patel S., Khanna D., Parmar M.S. (2021). Current pharmacological approaches and potential future therapies for Celiac disease. Eur. J. Pharmacol..

[75] Pais W.P., Duerksen D.R., Pettigrew N.M., Bernstein C.N. (2008). How many duodenal biopsy specimens are required to make a diagnosis of celiac disease?. Gastrointest. Endosc..

[76] Oberhuber G., Granditsch G., Vogelsang H. (1999). The histopathology of coeliac disease: Time for a standardized report scheme for pathologists. Eur. J. Gastroenterol. Hepatol..

[77] Corazza G.R., Villanacci V., Zambelli C., Milione M., Luinetti O., Vindigni C., Chioda C., Albarello L., Bartolini D., Donato F. (2007). Comparison of the interobserver reproducibility with different histologic criteria used in celiac disease. Clin. Gastroenterol. Hepatol..

[78] Catassi C., Fasano A. (2010). Celiac disease diagnosis: Simple rules are better than complicated algorithms. Am. J. Med..

[79] Raiteri A., Granito A., Giamperoli A., Catenaro T., Negrini G., Tovoli F. (2022). Current guidelines for the management of celiac disease: A systematic review with comparative analysis. World J. Gastroenterol..

[80] Husby S., Koletzko S., Korponay-Szabo I.R., Mearint M.L., Phillips A., Shamir R., Troncone R., Giersiepen K., Branski D., Catassi C. (2012). European Society for Pediatric Gastroenterology, Hepatology, and Nutrition guidelines for the diagnosis of coeliac disease. J. Pediatr. Gastroenterol. Nutr..

[81] Calado J., Machado M.V. (2021). Celiac Disease Revisited. GE Port. J. Gastroenterol..

[82] Wysocka-Mincewicz M., Groszek A., Ambrozkiewicz F., Paziewska A., Dąbrowska M., Rybak A., Konopka E., Ochocińska A., Żeber-Lubecka N., Karczmarski J. (2022). Combination of HLA-DQ2/-DQ8 Haplotypes and a Single MSH5 Gene Variant in a Polish Population of Patients with Type 1 Diabetes as a First Line Screening for Celiac Disease?. J. Clin. Med..

[83] Valitutti F., Trovato C.M., Montuori M., Cucchiara S. (2017). Pediatric celiac disease: Follow-up in the spotlight. Adv. Nutr..

[84] Bascuñán K.A., Vespa M.C., Araya M. (2017). Celiac disease: Understanding the gluten-free diet. Eur. J. Nutr..

[85] Fueyo-Díaz R., Montoro M., Magallón-Botaya R., Gascón-Santos S., Asensio-Martínez Á., Palacios-Navarro G., Sebastián-Domingo J.J. (2020). Influence of Compliance to Diet and Self-Efficacy Expectation on Quality of Life in Patients with Celiac Disease in Spain. Nutrients.

[86] Wagner G., Berger G., Sinnreich U., Grylli V., Schober E., Huber W.D., Karwautz A. (2008). Quality of life in adolescents with treated coeliac disease: Influence of compliance and age at diagnosis. J. Pediatr. Gastroenterol. Nutr..

[87] Dowhaniuk J.K., Mileski H., Saab J., Tutelman P., Thabane L., Brill H. (2020). The Gluten Free Diet: Assessing Adherence in a Pediatric Celiac Disease Population. J. Can. Assoc. Gastroenterol..

[88] Wieser H., Ruiz-Carnicer Á., Segura V., Comino I., Sousa C. (2021). Challenges of Monitoring the Gluten-Free Diet Adherence in the Management and Follow-Up of Patients with Celiac Disease. Nutrients.

[89] Stasi E., Marafini I., Caruso R., Soderino F., Angelucci E., Del Vecchio Blanco G., Paoluzi O.A., Calabrese E., Sedda S., Zorzi F. (2016). Frequency and Cause of Persistent Symptoms in Celiac Disease Patients on a Long-term Gluten-free Diet. J. Clin. Gastroenterol..

[90] Truitt K.E., Daveson A.J.M., Ee H.C., Goel G., MacDougall J., Neff K., Anderson R.P. (2019). Randomised clinical trial: A placebo-controlled study of subcutaneous or intradermal NEXVAX2, an investigational immunomodulatory peptide therapy for coeliac disease. Aliment. Pharmacol. Ther..

[91] Rubio-Tapia A., Kelly D.G., Lahr B.D., Dogan A., Wu T.T., Murray J.A. (2009). Clinical staging and survival in refractory celiac disease: A single center experience. Gastroenterology.

[92] Galli G., Carabotti M., Pilozzi E., Lahner E., Annibale B., Conti L. (2021). Relationship between Persistent Gastrointestinal Symptoms and Duodenal Histological Findings after Adequate Gluten-Free Diet: A Gray Area of Celiac Disease Management in Adult Patients. Nutrients.

[93] Sparks B., Hill I., Ediger T. (2021). Going Beyond Gluten-Free: A Review of Potential Future Therapies for Celiac Disease. Curr. Treat. Options Pediatr..

[94] Syage J.A., Green P.H.R., Khosla C., Adelman D.C., Sealey-Voyksner J.A., Murray J.A. (2019). Latiglutenase Treatment for Celiac Disease: Symptom and Quality of Life Improvement for Seropositive Patients on a Gluten-Free Diet. GastroHep.

[95] Calitri C., Fumi I., Ignaccolo M.G., Banino E., Benetti S., Lupica M.M., Fantone F., Pace M., Garofalo F. (2021). Gastrointestinal involvement in paediatric COVID-19-from pathogenesis to clinical management: A comprehensive review. World J. Gastroenterol..

[96] Cakir M., Guven B., Issi F., Ozkaya E. (2022). New-onset celiac disease in children during COVID-19 pandemic. Acta Paediatr..

[97] Trovato C.M., Montuori M., Pietropaoli N., Oliva S. (2021). COVID-19 and celiac disease: A pathogenetic hypothesis for a celiac outbreak. Int. J. Clin. Pract..

[98] Asri N., Nazemalhosseini Mojarad E., Mirjalali H., Mohebbi S.R., Baghaei K., Rostami-Nejad M., Yadegar A., Rezaei-Tavirani M., Asadzadeh Aghdaei H., Rostami K. (2021). Toward finding the difference between untreated celiac disease and COVID-19 infected patients in terms of CD4, CD25 (IL-2 Rα), FOXP3 and IL-6 expressions as genes affecting immune homeostasis. BMC Gastroenterol..

[99] Renzo S., Scarallo L., Antoniello L.M., Bramuzzo M., Chiaro A., Cisarò F., Contini A.C.I., De Angelis G.L., Angelis P., Nardo G.D. (2022). Impact of COVID-19 pandemic on pediatric endoscopy: A multicenter study on behalf of the SIGENP Endoscopy Working Group. Dig. Liver Dis..

[100] Concas G., Barone M., Francavilla R., Cristofori F., Dargenio V.N., Giorgio R., Dargenio C., Fanos V., Marcialis M.A. (2022). Twelve Months with COVID-19: What Gastroenterologists Need to Know. Dig. Dis. Sci..

[101] Uche-Anya E., Husby S., Kaplan G.G., Underwood F.E., Green P.H.R., Lebwohl B. (2021). An International Reporting Registry of Patients With Celiac Disease and COVID-19: Initial Results From SECURE-CELIAC. Clin. Gastroenterol. Hepatol..

[102] Mehtab W., Chauhan A., Agarwal A., Singh A., Rajput M.S., Mohta S., Jindal V., Banyal V., Ahmed A., Pramanik A. (2021). Impact of Corona Virus Disease 2019 pandemic on adherence to gluten-free diet in Indian patients with celiac disease. Indian J. Gastroenterol..

[103] Falcomer A.L., Farage P., Pratesi C.B., Pratesi R., Gandolfi L., Nakano E.Y., Raposo A., Zandonadi R.P. (2021). Health-Related Quality of Life and Experiences of Brazilian Celiac Individuals over the Course of the Sars-Cov-2 Pandemic. Nutrients.

[104] Monzani A., Lionetti E., Felici E., Fransos L., Azzolina D., Rabbone I., Catassi C. (2020). Adherence to the Gluten-Free Diet during the Lockdown for COVID-19 Pandemic: A Web-Based Survey of Italian Subjects with Celiac Disease. Nutrients.

[105] Temsah M.H., Aljamaan F., Alhaboob A., Almosned B., Alsebail R., Temsah R., Senjab A., Alarfaj A., Aljudi T., Jamal A. (2022). Enhancing parental knowledge of childhood and adolescence safety: An interventional educational campaign. Medicine.

[106] Barschkett M., Koletzko B., Spiess C.K. (2021). COVID-19 Associated Contact Restrictions in Germany: Marked Decline in Children’s Outpatient Visits for Infectious Diseases without Increasing Visits for Mental Health Disorders. Children.

[107] Dipasquale V., Passanisi S., Cucinotta U., Cascio A., Romano C. (2021). Implications of SARS-COV-2 infection in the diagnosis and management of the pediatric gastrointestinal disease. Ital. J. Pediatr..

[108] Bükülmez A., Baş M.T., Çiftci E. (2021). Evaluation of anti-COVID-19 measures taken by the parents of children with celiac disease: A cross-sectional study. Sao Paulo Med. J..

[109] Lionetti E., Fabbrizi A., Catassi C. (2021). Prevalence of COVID-19 in Italian Children With Celiac Disease: A Cross-Sectional Study. Clin. Gastroenterol. Hepatol..

[110] Catassi G.N., Vallorani M., Cerioni F., Lionetti E., Catassi C. (2020). A negative fallout of COVID-19 lockdown in Italy: Life-threatening delay in the diagnosis of celiac disease. Dig. Liver. Dis..

[111] Feldstein L.R., Tenforde M.W., Friedman K.G., Newhams M., Rose E.B., Dapul H., Soma V.L., Maddux A.B., Mourani P.M., Bowens C. (2021). Overcoming COVID-19 Investigators. Characteristics and Outcomes of US Children and Adolescents With Multisystem Inflammatory Syndrome in Children (MIS-C) Compared With Severe Acute COVID-19. JAMA.

[112] Fremed M.A., Farooqi K.M. (2022). Longitudinal Outcomes and Monitoring of Patients With Multisystem Inflammatory Syndrome in Children. Front. Pediatr..

[113] Belhadjer Z., Méot M., Bajolle F., Khraiche D., Legendre A., Abakka S., Auriau J., Grimaud M., Oualha M., Beghetti M. (2020). Acute Heart Failure in Multisystem Inflammatory Syndrome in Children in the Context of Global SARS-CoV-2 Pandemic. Circulation.

[114] Feldstein L.R., Rose E.B., Horwitz S.M., Collins J.P., Newhams M.M., Son M.B.F., Newburger J.W., Kleinman L.C., Heidemann S.M., Martin A.A. (2020). Multisystem Inflammatory Syndrome in U.S. Children and Adolescents. N. Engl. J. Med..

[115] Cheung E.W., Zachariah P., Gorelik M., Boneparth A., Kernie S.G., Orange J.S., Milner J.D. (2020). Multisystem Inflammatory Syndrome Related to COVID-19 in Previously Healthy Children and Adolescents in New York City. JAMA.

[116] Nakra N.A., Blumberg D.A., Herrera-Guerra A., Lakshminrusimha S. (2020). Multi-System Inflammatory Syndrome in Children (MIS-C) Following SARS-CoV-2 Infection: Review of Clinical Presentation, Hypothetical Pathogenesis, and Proposed Management. Children.

[117] Cattalini M., Della Paolera S., Zunica F., Bracaglia C., Giangreco M., Verdoni L., Meini A., Sottile R., Caorsi R., Zuccotti G. (2021). Defining Kawasaki disease and pediatric inflammatory multisystem syndrome-temporally associated to SARS-CoV-2 infection during SARS-CoV-2 epidemic in Italy: Results from a national, multicenter survey. Pediatr. Rheumatol..

[118] Consiglio C.R., Cotugno N., Sardh F., Pou C., Amodio D., Rodriguez L., Tan Z., Zicari S., Ruggiero A., Pascucci G.R. (2020). The Immunology of Multisystem Inflammatory Syndrome in Children with COVID-19. Cell.

[119] Pfeifer J., Thurner B., Kessel C., Fadle N., Kheiroddin P., Regitz E., Hoffmann M.C., Kos I.A., Preuss K.D., Fischer Y. (2022). Autoantibodies against interleukin-1 receptor antagonist in multisystem inflammatory syndrome in children: A multicentre, retrospective, cohort study. Lancet Rheumatol..

[120] Dhaliwal M., Tyagi R., Malhotra P., Barman P., Loganathan S.K., Sharma J., Sharma K., Mondal S., Rawat A., Singh S. (2022). Mechanisms of Immune Dysregulation in COVID-19 Are Different From SARS and MERS: A Perspective in Context of Kawasaki Disease and MIS-C. Front. Pediatr..

[121] Gruber C., Patel R., Trachman R., Lepow L., Amanat F., Krammer F., Wilson K.M., Onel K., Geanon D., Tuballes K. (2020). Mapping Systemic Inflammation and Antibody Responses in Multisystem Inflammatory Syndrome in Children (MIS-C). Cell.

[122] Diorio C., Henrickson S.E., Vella L.A., McNerney K.O., Chase J., Burudpakdee C., Lee J.H., Jasen C., Balamuth F., Barrett D.M. (2020). Multisystem inflammatory syndrome in children and COVID-19 are distinct presentations of SARS-CoV-2. J. Clin. Investig..

[123] Bartsch Y.C., Wang C., Zohar T., Fischinger S., Atyeo C., Burke J.S., Kang J., Edlow A.G., Fasano A., Baden L.R. (2021). Humoral signatures of protective and pathological SARS-CoV-2 infection in children. Nat. Med..

[124] Yonker L.M., Gilboa T., Ogata A.F., Senussi Y., Lazarovits R., Boribong B.P., Bartsch Y.C., Loiselle M., Rivas M.N., Porritt R.A. (2021). Multisystem inflammatory syndrome in children is driven by zonulin-dependent loss of gut mucosal barrier. J. Clin. Investig..

[125] Fasano A., Not T., Wang W., Uzzau S., Berti I., Tommasini A., Goldblum S.E. (2000). Zonulin, a newly discovered modulator of intestinal permeability, and its expression in coeliac disease. Lancet.

[126] Ayabe T., Satchell D.P., Wilson C.L., Parks W.C., Selsted M.E., Ouellette A.J. (2000). Secretion of microbicidal alpha-defensins by intestinal Paneth cells in response to bacteria. Nat Immunol..

[127] Ayabe T., Ashida T., Kohgo Y., Kono T. (2004). The role of Paneth cells and their antimicrobial peptides in innate host defense. Trends Microbiol..

[128] Stolfi C., Maresca C., Monteleone G., Laudisi F. (2022). Implication of Intestinal Barrier Dysfunction in Gut Dysbiosis and Diseases. Biomedicines.

[129] Heyman M., Abed J., Lebreton C., Cerf-Bensussan N. (2012). Intestinal permeability in coeliac disease: Insight into mechanisms and relevance to pathogenesis. Gut.

[130] Cardoso-Silva D., Delbue D., Itzlinger A., Moerkens R., Withoff S., Branchi F., Schumann M. (2019). Intestinal Barrier Function in Gluten-Related Disorders. Nutrients.

[131] Günzel D., Yu A.S. (2013). Claudins and the modulation of tight junction permeability. Physiol. Rev..

[132] Chelakkot C., Ghim J., Ryu S.H. (2018). Mechanisms regulating intestinal barrier integrity and its pathological implications. Exp. Mol. Med..

[133] Hartsock A., Nelson W.J. (2008). Adherens and tight junctions: Structure, function and connections to the actin cytoskeleton. Biochim. Biophys. Acta.

[134] Wood Heickman L.K., DeBoer M.D., Fasano A. (2020). Zonulin as a potential putative biomarker of risk for shared type 1 diabetes and celiac disease autoimmunity. Diabetes/Metab. Res. Rev..

[135] Jian C., Kanerva S., Qadri S., Yki-Järvinen H., Salonen A. (2022). In vitro Effects of Bacterial Exposure on Secretion of Zonulin Family Peptides and Their Detection in Human Tissue Samples. Front. Microbiol..

[136] Hashimoto Y., Campbell M. (2020). Tight junction modulation at the blood-brain barrier: Current and future perspectives. Biochim. Biophys. Acta (BBA)–Biomembr..

[137] Di Pierro M., Lu R., Uzzau S., Wang W., Margaretten K., Pazzani C., Maimone F., Fasano A. (2001). Zonula occludens toxin structure-function analysis. Identification of the fragment biologically active on tight junctions and of the zonulin receptor binding domain. J. Biol. Chem..

[138] Fasano A. (2011). Zonulin and its regulation of intestinal barrier function: The biological door to inflammation, autoimmunity, and cancer. Physiol. Rev..

[139] Sturgeon C., Fasano A. (2016). Zonulin, a regulator of epithelial and endothelial barrier functions, and its involvement in chronic inflammatory diseases. Tissue Barriers.

[140] Martinez E.E., Lan J., Konno T., Miranda-Ribera A., Fiorentino M., Mehta N.M., Fasano A. (2021). Novel role of zonulin in the pathophysiology of gastro-duodenal transit: A clinical and translational study. Sci. Rep..

[141] Wang X., Memon A.A., Palmér K., Hedelius A., Sundquist J., Sundquist K. (2022). The association of zonulin-related proteins with prevalent and incident inflammatory bowel disease. BMC Gastroenterol..

[142] Tripathi A., Lammers K.M., Goldblum S., Shea-Donohue T., Netzel-Arnett S., Buzza M.S., Antalis T.M., Vogel S.N., Zhao A., Yang S. (2009). Identification of human zonulin, a physiological modulator of tight junctions, as prehaptoglobin-2. Proc. Natl. Acad. Sci. USA.

[143] Sturgeon C., Lan J., Fasano A., Ann N.Y. (2017). Zonulin transgenic mice show altered gut permeability and increased morbidity/mortality in the DSS colitis model. Acad. Sci..

[144] Mashaqi S., Kallamadi R., Matta A., Quan S.F., Patel S.I., Combs D., Estep L., Lee-Iannotti J., Smith C., Parthasarathy S. (2022). Obstructive Sleep Apnea as a Risk Factor for COVID-19 Severity—The Gut Microbiome as a Common Player Mediating Systemic Inflammation via Gut Barrier Dysfunction. Cells.

[145] Yonker L.M., Swank Z., Gilboa T., Senussi Y., Kenyon V., Papadakis L., Boribong B.P., Carroll R.W., Walt D.R., Fasano A. (2022). Zonulin Antagonist, Larazotide (AT1001), As an Adjuvant Treatment for Multisystem Inflammatory Syndrome in Children: A Case Series. Crit. Care Explor..

[146] American Academy of Pediatrics/Home/Children and COVID-19: State-Level Data Report. https://www.aap.org/en/pages/2019-novel-coronavirus-covid-19-infections/children-and-covid-19-state-level-data-report/.

[147] Centers for Disease Control and Prevention (CDC) COVID Data Tracker. Health Department-Reported Cases of Multisystem Inflammatory Syndrome in Children (MIS-C) in the United States. https://covid.cdc.gov/covid-data-tracker/#mis-national-surveillance.

[148] Abrams J.Y., Godfred-Cato S.E., Oster M.E., Chow E.J., Koumans E.H., Bryant B., Leung J.W., Belay E.D. (2020). Multisystem Inflammatory Syndrome in Children Associated with Severe Acute Respiratory Syndrome Coronavirus 2: A Systematic Review. J. Pediatr..

[149] Mao R., Qiu Y., He J.S., Tan J.Y., Li X.H., Liang J., Shen J., Zhu L.R., Chen Y., Iacucci M. (2020). Manifestations and prognosis of gastrointestinal and liver involvement in patients with COVID-19: A systematic review and meta-analysis. Lancet Gastroenterol. Hepatol..

[150] Sultan S., Altayar O., Siddique S.M., Davitkov P., Feuerstein J.D., Lim J.K., Falck-Ytter Y., El-Serag H.B. (2020). AGA Institute Rapid Review of the Gastrointestinal and Liver Manifestations of COVID-19, Meta-Analysis of International Data, and Recommendations for the Consultative Management of Patients with COVID-19. Gastroenterology.

[151] Bankaitis E.D., Ha A., Kuo C.J., Magness S.T. (2018). Reserve stem cells in intestinal homeostasis and injury. Gastroenterology.

[152] Belcher J.D., Zhang P., Nguyen J., Kiser Z.M., Nath K.A., Hu J., Trent J.O., Vercellotti G.M. (2020). Identification of a Heme Activation Site on the MD-2/TLR4 Complex. Front. Immunol..

[153] Santaolalla R., Abreu M.T. (2012). Innate immunity in the small intestine. Curr. Opin. Gastroenterol..

[154] Alibeik N., Pishgar E., Bozorgmehr R., Aghaaliakbari F., Rahimian N. (2022). Potential role of gut microbiota in patients with COVID-19, its relationship with lung axis, central nervous system (CNS) axis, and improvement with probiotic therapy. Iran. J. Microbiol..

[155] Llorens S., Nava E., Muñoz-López M., Sánchez-Larsen Á., Segura T. (2021). Neurological Symptoms of COVID-19: The Zonulin Hypothesis. Front. Immunol..

[156] Giron L.B., Dweep H., Yin X., Wang H., Damra M., Goldman A.R., Gorman N., Palmer C.S., Tang H.Y., Shaikh M.W. (2021). Plasma Markers of Disrupted Gut Permeability in Severe COVID-19 Patients. Front. Immunol..

[157] Trottein F., Sokol H. (2020). Potential Causes and Consequences of Gastrointestinal Disorders during a SARS-CoV-2 Infection. Cell Rep..

[158] Campisi L., Barbet G., Ding Y., Esplugues E., Flavell R.A., Blander J.M. (2016). Apoptosis in response to microbial infection induces autoreactive TH17 cells. Nat. Immunol..

[159] Hensley-McBain T., Manuzak J.A. (2021). Zonulin as a biomarker and potential therapeutic target in multisystem inflammatory syndrome in children. J. Clin. Investig..

[160] Mønsted M.Ø., Falck N.D., Pedersen K., Buschard K., Holm L.J., Haupt-Jorgensen M. (2021). Intestinal permeability in type 1 diabetes: An updated comprehensive overview. J. Autoimmun..

[161] Barker J.M., Liu E. (2008). Celiac disease: Pathophysiology, clinical manifestations, and associated autoimmune conditions. Adv. Pediatr..

[162] del Servicio Canario S.D.E., de la Salud (SESCS) (2018). Protocolo Para El Diagnóstico Precoz de la Enfermedad Celíaca. https://www.sanidad.gob.es/profesionales/prestacionesSanitarias/publicaciones/Celiaquia/enfermedadCeliaca.pdf.

[163] Lebeaux R.M., Madan J.C., Nguyen Q.P., Coker M.O., Dade E.F., Moroishi Y., Palys T.J., Ross B.D., Pettigrew M.M., Morrison H.G. (2022). Impact of antibiotics on off-target infant gut microbiota and resistance genes in cohort studies. Pediatr. Res..

[164] Dydensborg Sander S., Nybo Andersen A.M., Murray J.A., Karlstad Ø., Husby S., Størdal K. (2019). Association between antibiotics in the first year of life and celiac disease. Gastroenterology.

[165] Caminero A., Verdu E.F. (2019). Celiac disease: Should we care about microbes?. Am. J. Physiol. Gastrointest. Liver Physiol..

[166] Verdu E.F., Schuppan D. (2021). Co-factors, Microbes, and Immunogenetics in Celiac Disease to Guide Novel Approaches for Diagnosis and Treatment. Gastroenterology.

[167] Alavi Moghaddam M., Rostami Nejad M., Shalmani H.M., Rostami K., Nazemalhosseini Mojarad E., Aldulaimi D., Zali M.R. (2013). The effects of gluten-free diet on hypertransaminasemia in patients with celiac disease. Int. J. Prev. Med..

[168] Mostarica-Stojković M. (2005). Mechanisms of the induction of autoimmunity. Srp. Arh. za Celok. Lek..

[169] Tang D., Kang R., Coyne C.B., Zeh H.J., Lotze M.T. (2012). PAMPs and DAMPs: Signal 0s that spur autophagy and immunity. Immunol. Rev..

[170] Bai X., Shi H., Yang M., Wang Y., Sun Z., Xu S. (2018). Identification of key genes implicated in the suppressive function of human FOXP3+CD25+CD4+ regulatory T cells through the analysis of time-series data. Mol. Med. Rep..

[171] Barartabar Z., Nikzamir A., Sirati-Sabet M., Aghamohammadi E., Chaleshi V., Rostami Nejad M., Asadzadeh-Aghdaei H., Reza Z.M. (2018). The relationship between 174 G/C and -572 G/C of IL-6 gene polymorphisms and susceptibility of celiac disease in the Iranian population. Prz. Gastroenterol..

[172] Nasserinejad M., Shojaee S., Ghobakhlou M., Lak E., Eslami P., Pourhoseingholi M.A. (2019). The effects of IL-8, IL- 6, and IL-1 on the risk of celiac disease: A Bayesian regression analysis. Gastroenterol. Hepatol. Bed Bench.

[173] Grifoni E., Valoriani A., Cei F., Lamanna R., Gelli A.M.G., Ciambottit B., Vannucchi V., Moroni F., Pelagatti L., Tarquini R. (2020). Interleukin-6 as prognosticator in patients with COVID-19. J. Infect..

[174] Nasonov E., Samsonov M. (2020). The role of Interleukin 6 inhibitors in therapy of severe COVID-19. Biomed. Pharmacother..

[175] Mojtabavi H., Saghazadeh A., Rezaei N. (2020). Interleukin-6 and severe COVID-19: A systematic review and meta-analysis. Eur. Cytokine Netw..

[176] Wang C., Fei D., Li X., Zhao M., Yu K. (2020). IL-6 may be a good biomarker for earlier detection of COVID-19 progression. Intensive Care Med..

[177] Ouyang W., Kolls J.K., Zheng Y. (2008). The biological functions of T helper 17 cell effector cytokines in inflammation. Immunity.

[178] Shekhawat J., Gauba K., Gupta S., Purohit P., Mitra P., Garg M., Misra S., Sharma P., Banerjee M. (2021). Interleukin-6 Perpetrator of the COVID-19 Cytokine Storm. Indian J. Clin. Biochem..

[179] Pecora F., Persico F., Gismondi P., Fornaroli F., Iuliano S., de’Angelis G.L. (2020). Esposito, S. Gut Microbiota in Celiac Disease: Is There Any Role for Probiotics?. Front. Immunol..

[180] Mårild K., Fredlund H., Ludvigsson J.F. (2010). Increased risk of hospital admission for influenza in patients with celiac disease: A nationwide cohort study in Sweden. Am. J. Gastroenterol..

[181] Magazzù G., Aquilina S., Barbara C., Bondin R., Brusca I., Bugeja J., Camilleri M., Cascio D., Costa S., Cuzzupè C. (2022). Recognizing the Emergent and Submerged Iceberg of the Celiac Disease: ITAMA Project—Global Strategy Protocol. Pediatr. Rep..

[182] Elli L., Barisani D., Vaira V., Bardella M.T., Topa M., Vecchi M., Doneda L., Scricciolo A., Lombardo V., Roncoroni L. (2020). How to manage celiac disease and gluten-free diet during the COVID-19 era: Proposals from a tertiary referral center in a high-incidence scenario. BMC Gastroenterol..

[183] Hadi Y.B., Sohail A.H., Lakhani D.A., Naqvi S.F., Kupec J.T., Pervez A. (2022). Outcomes of SARS-CoV-2 infection in patients with celiac disease: A multicenter research network study. Ann. Gastroenterol..

[184] Greco N., Meacci A., Mora B., Vestri A., Picarelli A. (2022). Coeliac disease in the COVID-19 pandemic: Does HLA have a protective effect?. Ann. Med..

[185] Samasca G., Lerner A. (2021). Celiac disease in the COVID-19 pandemic. J. Transl. Autoimmun..

[186] Lefthériotis G., Wray S., Girardi A.C.C., Vidal-Petiot E., Bailey M.A., Schechtman D., Ravi N., Noble D. (2021). Editorial: The Tribute of Physiology for the Understanding of COVID-19 Disease. Front. Physiol..

[187] Ailioaie L.M., Ailioaie C., Litscher G. (2022). Implications of SARS-CoV-2 Infection in Systemic Juvenile Idiopathic Arthritis. Int. J. Mol. Sci..

[188] Costantino A., Topa M., Roncoroni L., Doneda L., Lombardo V., Stocco D., Gramegna A., Costantino C., Vecchi M., Elli L. (2021). COVID-19 Vaccine: A Survey of Hesitancy in Patients with Celiac Disease. Vaccines.

[189] Zhen J., Stefanolo J.P., Temprano M.P., Seiler C.L., Caminero A., de-Madaria E., Huguet M.M., Santiago V., Niveloni S.I., Smecuol E.G. (2021). Risk perception and knowledge of COVID-19 in patients with celiac disease. World J. Gastroenterol..

[190] Aaron Lerner (2021). The COVID-19 Vaccination Debate: CoV-2 in Celiac Disease: A Pathogen or just along for the Ride?. Int. J. Celiac Dis..

[191] Cascini F., Pantovic A., Al-Ajlouni Y., Failla G., Ricciardi W. (2021). Attitudes, acceptance and hesitancy among the general population worldwide to receive the COVID-19 vaccines and their contributing factors: A systematic review. eClinicalMedicine.

